# The Protein-Protein Interaction Network Reveals a Novel Role of the Signal Transduction Protein PII in the Control of c-di-GMP Homeostasis in Azospirillum brasilense

**DOI:** 10.1128/mSystems.00817-20

**Published:** 2020-11-03

**Authors:** Edileusa C. M. Gerhardt, Erick Parize, Fernanda Gravina, Flávia L. D. Pontes, Adrian R. S. Santos, Gillize A. T. Araújo, Ana C. Goedert, Alysson H. Urbanski, Maria B. R. Steffens, Leda S. Chubatsu, Fabio O. Pedrosa, Emanuel M. Souza, Karl Forchhammer, Elena Ganusova, Gladys Alexandre, Gustavo A. de Souza, Luciano F. Huergo

**Affiliations:** a Departamento de Bioquímica e Biologia Molecular, UFPR Curitiba, Curitiba, PR, Brazil; b Interfakultäres Institut für Mikrobiologie und Infektionsmedizin der Eberhard-Karls Universität Tübingen, Tübingen, Germany; c Department of Biochemistry, Cellular & Molecular Biology, The University of Tennessee, Knoxville, Tennessee, USA; d Departamento de Bioquímica, Universidade Federal do Rio Grande do Norte, Natal, RN, Brazil; e Bioinformatics Multidisciplinary Environment, UFRN, Natal, Brazil; f Setor Litoral, UFPR Matinhos, Curitiba, PR, Brazil; State University of Maringá

**Keywords:** protein interaction, PII protein, c-di-GMP, motility, metabolic regulation, cell motility, metabolome, protein interactions

## Abstract

The PII family comprises a group of widely distributed signal transduction proteins ubiquitous in prokaryotes and in the chloroplasts of plants. PII proteins sense the levels of key metabolites ATP, ADP, and 2-oxoglutarate, which affect the PII protein structure and thereby the ability of PII to interact with a range of target proteins. Here, we performed multiple ligand fishing assays with the PII protein orthologue GlnZ from the plant growth-promoting nitrogen-fixing bacterium Azospirillum brasilense to identify 37 proteins that are likely to be part of the PII protein-protein interaction network. Among the PII targets identified were enzymes related to nitrogen and fatty acid metabolism, signaling, coenzyme synthesis, RNA catabolism, and transcription. Direct binary PII-target complex was confirmed for 15 protein complexes using pulldown assays with recombinant proteins. Untargeted metabolome analysis showed that PII is required for proper homeostasis of important metabolites. Two enzymes involved in c-di-GMP metabolism were among the identified PII targets. A PII-deficient strain showed reduced c-di-GMP levels and altered aerotaxis and flocculation behavior. These data support that PII acts as a major metabolic hub controlling important enzymes and the homeostasis of key metabolites such as c-di-GMP in response to the prevailing nutritional status.

**IMPORTANCE** The PII proteins sense and integrate important metabolic signals which reflect the cellular nutrition and energy status. Such extraordinary ability was capitalized by nature in such a way that the various PII proteins regulate different facets of metabolism by controlling the activity of a range of target proteins by protein-protein interactions. Here, we determined the PII protein interaction network in the plant growth-promoting nitrogen-fixing bacterium Azospirillum brasilense. The interactome data along with metabolome analysis suggest that PII functions as a master metabolic regulator hub. We provide evidence that PII proteins act to regulate c-di-GMP levels *in vivo* and cell motility and adherence behaviors.

## Abstract (precis)

The PII proteins sense and integrate important metabolic signals which reflect the cellular nutrition and energy status. Such extraordinary ability was capitalized by nature in such a way that the various PII proteins regulate different facets of metabolism by controlling the activity of a range of target proteins by protein-protein interactions. Here, we determined the PII protein interaction network in the plant growth-promoting nitrogen-fixing bacterium Azospirillum brasilense. The interactome data along with metabolome analysis suggest that PII functions as a master metabolic regulator hub. We provide evidence that PII proteins act to regulate c-di-GMP levels *in vivo* and cell motility and adherence behaviors.

## INTRODUCTION

Limitation of nutrients affects the growth and fitness of prokaryotes. The ability to reproduce and compete in natural environments requires sophisticated mechanisms to sense nutrient availability and to tune key metabolic pathways accordingly. One of the most ubiquitous sensory systems present in almost all prokaryotes and in the chloroplasts of Archaeplastida is the PII signaling proteins (1, 2). The PII proteins are likely to have evolved in a deep-branching prokaryotic life form and were conserved during the evolution of *Bacteria*, *Archaea*, and chloroplasts.

The role of PII proteins in signaling was for the first time observed in Escherichia coli due to the ability to regulate the activity of glutamine synthetase adenylyltransferase (ATase) (EC 2.7.7.49), an enzyme involved in the regulation of glutamine synthetase activity (3). Since this pioneering study, others have shown that PII proteins act as a widespread regulator of nitrogen and, more recently, carbon metabolism, in a diverse range of prokaryotes, including *Archaea* (4). The sensory properties of canonical PII proteins rely on their ability to sense the intracellular levels of key metabolites ATP, ADP, and 2-oxoglutarate (2-OG). PII proteins can also sense l-glutamine either by allosteric binding or indirectly via reversible posttranslational modification (5).

Canonical PII proteins are very conserved in sequence and three-dimensional structure. They form homotrimers with a compact barrel-like structure with a flexible loop, namely, T-loop, which emerges from each subunit (1). PII proteins have three nucleotide-binding sites located at the lateral clefts between each subunit (6) which can be competitively occupied by ATP or ADP, thereby rendering PII able to sense the energy status as reported by the ATP/ADP ratio (7–10). Another key PII allosteric effector is 2-OG, a tricarboxylic acid (TCA) cycle intermediate used as a carbon skeleton for nitrogen assimilation reactions. The 2-OG levels act as a proxy of the balance between nitrogen and carbon availability (11). The three 2-OG binding sites are located within the vicinity of the nucleotide-binding sites, and the 2-OG allosteric site is formed upon previous MgATP binding (12, 13). Hence, 2-OG and MgATP binding are synergistic, while 2-OG and ADP binding are antagonistic (14). Such a mechanism allows PII to integrate cell energy levels (ATP/ADP ratio) with carbon to nitrogen availability (2-OG levels).

Additional signal input for PII came from l-glutamine, a metabolite whose levels act as an indicator of nitrogen availability in *Bacteria* (15). In *Proteobacteria*, l-glutamine sensing requires an additional protein, namely, GlnD, which coordinates PII reversible posttranslational modification accordingly to the prevailing l-glutamine levels (16). Quite remarkably, in plants, l-glutamine sensing occurs through a unique PII C-terminal extension which allosterically binds glutamine (17)

In *Bacteria*, *Archaea*, and chloroplasts, PII is engaged in regulating nitrogen and carbon metabolic pathways by interacting with key cellular proteins, including transporters, enzymes, and transcriptional regulators (1). These PII protein-protein interactions are controlled by conformational changes in the PII structure caused by allosteric effector binding and posttranslational modification. In general, the binding of 2-OG and posttranslational modification induce a PII T-loop conformation which abrogates most PII-protein interactions (5).

Proteobacteria frequently encode multiple PII protein paralogues, which may have overlapping but also specific functions (18). For example, the plant growth-promoting nitrogen-fixing bacterium Azospirillum brasilense encodes two PII paralogues, namely, GlnB and GlnZ (19). While both proteins can interact with the ammonium transporter AmtB (20), only GlnB interacts with the nitrogenase regulatory proteins NifA and DraT (21, 22). On the other hand, GlnZ interacts preferentially with DraG (23). Both *glnB* and *glnZ* genes are under the control of the global Ntr system; thus, GlnB expression and GlnZ expression are induced under nitrogen limitation (19, 24).

Recent work in bacteria and plants revealed several novel PII targets that are not only involved in nitrogen metabolism, but in general core metabolism, such as acetyl-coenzyme A (CoA) carboxylase, phosphoenolpyruvate carboxylase (PEPC), NAD synthetase, and glucosamine 6-phosphate deaminase (25–27). Therefore, PII has a much broader regulatory role than originally thought. We have previously used PII affinity columns to successfully identify acetyl-CoA carboxylase and NAD synthetase as binding partners of the PII protein GlnZ in A. brasilense (26, 28). Here, we performed multiple GlnZ ligand fishing assays to identify 37 proteins that are likely to be part of the PII protein interaction network in *A. brasilense*. The data support that PII regulates a plethora of key metabolic pathways which includes c-di-GMP levels affecting chemotaxis in a gradient of air (aerotaxis) and flocculation behavior. This knowledge provides a framework for a better comprehension of metabolic regulation in *A. brasilense*, which can be exploited in the future to improve *A. brasilense* biofertilizer traits.

## RESULTS

### Identification of potential GlnZ-interacting proteins in *A. brasilense* extracts.

In order to provide a comprehensive analysis of the GlnZ interaction network in *A. brasilense*, we performed extensive ligand fishing assays. A range of GlnZ affinity columns were used, including His-tagged and FLAG-tagged GlnZ as baits (see Materials and Methods for details). These columns were challenged with total *A. brasilense* protein extracts in the presence of ATP or ADP and the potential GlnZ-binding proteins were eluted from these columns in buffer containing MgATP and 2-OG. The presence of 2-OG significantly alters the GlnZ protein structure (12) and affects the stability of GlnZ-target complexes, allowing the specific elution of proteins that were retained in the column by interaction with GlnZ. A total of six independent assays were considered for the analyses: in four assays, proteins were allowed to interact with GlnZ in the presence of ATP and were eluted in the presence of ATP plus 2-OG (assays named ATP1 to ATP4); in two assays, proteins were allowed to interact with GlnZ in the presence of ADP and were eluted in the presence of ATP plus 2-OG (assays named ADP1 and ADP2). Columns without GlnZ were used as negative controls to discriminate between specific GlnZ targets and unspecific background. The extracts from all these columns were analyzed by label-free liquid chromatography coupled to tandem mass spectrometry (LC-MS/MS). Only two replicate ADP assays were performed given that a lower number of proteins bound to PII were identified in comparison to the experiments performed in the presence of ATP.

Proteins were considered potential GlnZ-binding candidates when their relative abundances were ≥3-fold enriched with a *P* value of less than 0.05 in the fraction eluted from the GlnZ column in comparison to the respective control. Using these criteria, 86, 54, 55, 43, 15, or 16 proteins were selected in the assays ATP1, ATP2, ATP3, ATP4, ADP1, and ADP2, respectively (see Table S1 in the supplemental material). After dereplication, 118 proteins remained for further analysis, of which 9 were exclusively enriched in ADP assays. In order to provide a list of the most likely GlnZ-binding candidates, we further filtered proteins that were enriched in at least three out of the four ATP assays or in the two of the ADP assays. This resulted in a list of 37 proteins (Table 1) that are likely to be part of the GlnZ interaction network. The *N*-acetyl-l-glutamate kinase (NAGK) enzyme was enriched only in the ATP1 assay; however, it was considered for further analysis since it is a known PII-binding partner in other organisms (29). Samples from ATP assays were also subjected to sodium dodecyl sulfate-polyacrylamide gel electrophoresis (SDS-PAGE) analysis, and protein bands specifically enriched in the GlnZ affinity column samples were identified by in-gel trypsin digestion followed by matrix-assisted laser desorption ionization−time of flight (MALDI-TOF) peptide mass fingerprint analysis. This analysis further supports the enrichment of eight proteins among those selected by the LC-MS/MS approach (Table 1).

**TABLE 1:**
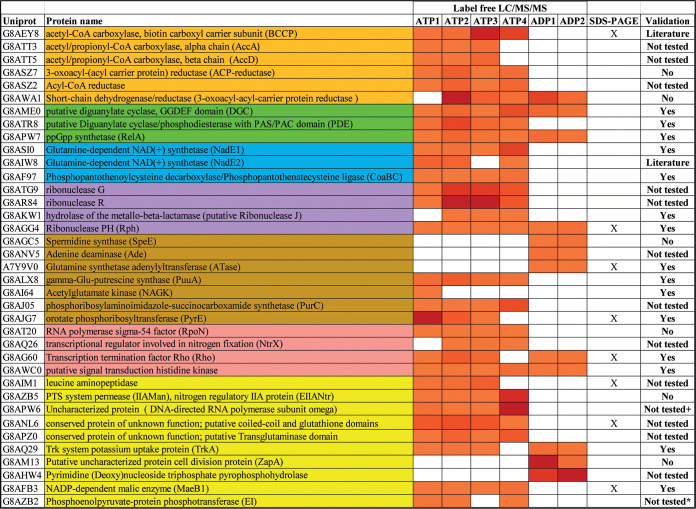
The *A. brasilense* GlnZ interaction network^*a*^

a The 37 proteins that were considered the GlnZ interaction network are separated accordingly to their predicted function: fatty acid metabolism (orange), secondary messenger metabolism (green), enzyme cofactor metabolism (blue), RNA catabolism (purple), nitrogen metabolism (brown), transcription (pink), and other function (yellow). The columns ATP1 to ATP4 and ADP1 and ADP2 represent LC-MS/MS label-free proteomic data from each GlnZ ligand fishing experiment. The relative protein enrichment in comparison to control is represented as a heat map, increasing from orange to dark red. Proteins that were considered enriched in the ATP assays as judged by visual inspection of SDS-PAGE gels followed by identification by peptide mass fingerprint are indicated with an X in the SDS-PAGE column. In the validation column, “Yes” indicates that protein complex formation with GlnZ was confirmed by pulldown analysis in the current study, “Literature” indicates confirmation of complex formation with PII protein in previous studies, “No” indicates negative results in pulldown analysis, and “Not tested” followed by a plus sign or by an asterisk indicates that the corresponding protein could not be overexpressed or was completely insoluble, respectively. PTS, phosphotransferase system.

10.1128/mSystems.00817-20.2 TABLE S1 Proteins enriched in GlnZ ligand fishing assays. The ATP1 to ATP4 and ADP1 and ADP2 columns indicate the code of each experiment. The number below each code shows the enrichment of each protein in relation to the control. In some cases, enrichment was confirmed by visual inspection of protein bands on SDS-PAGE following by protein identification (ID) using peptide mass fingerprint MALDI-TOF analysis. Positive protein IDs are marked with a X. Download Table S1, XLSX file, 0.04 MB. Copyright © 2020 Gerhardt et al. 2020 Gerhardt et al. This content is distributed under the terms of the Creative Commons Attribution 4.0 International license.

Several proteins related to fatty acid metabolism, nitrogen metabolism, signaling, coenzyme synthesis, RNA catabolism, and transcription were among the GlnZ-binding target candidates. As a proof of concept, previously described *A. brasilense* GlnZ-binding targets, BCCP (biotin carboxyl carrier protein) and NadE2, were among the predicted GlnZ target candidates, thereby validating our experimental design (Table 1).

### Validation of potential GlnZ-interacting proteins by pulldown.

**FIG 1:**
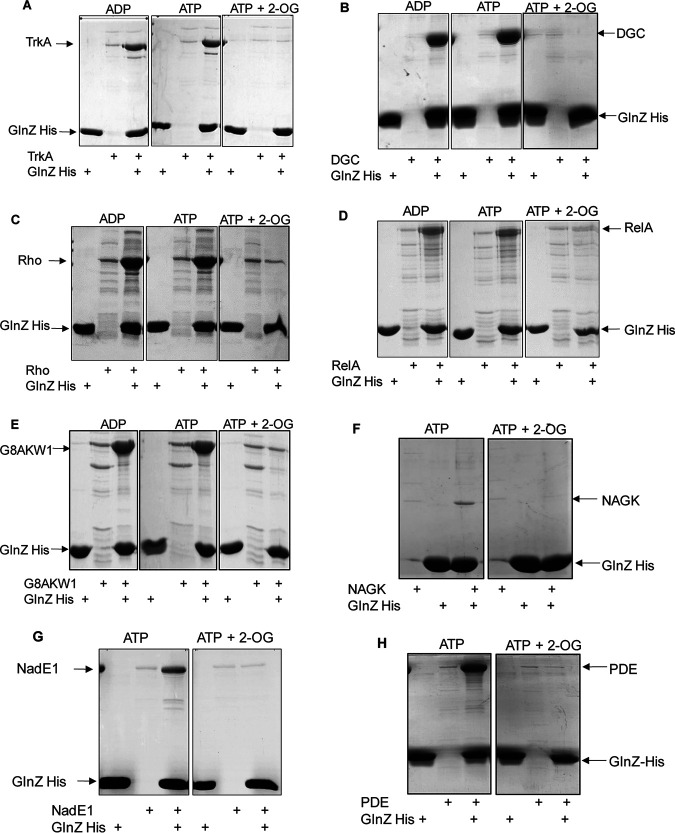

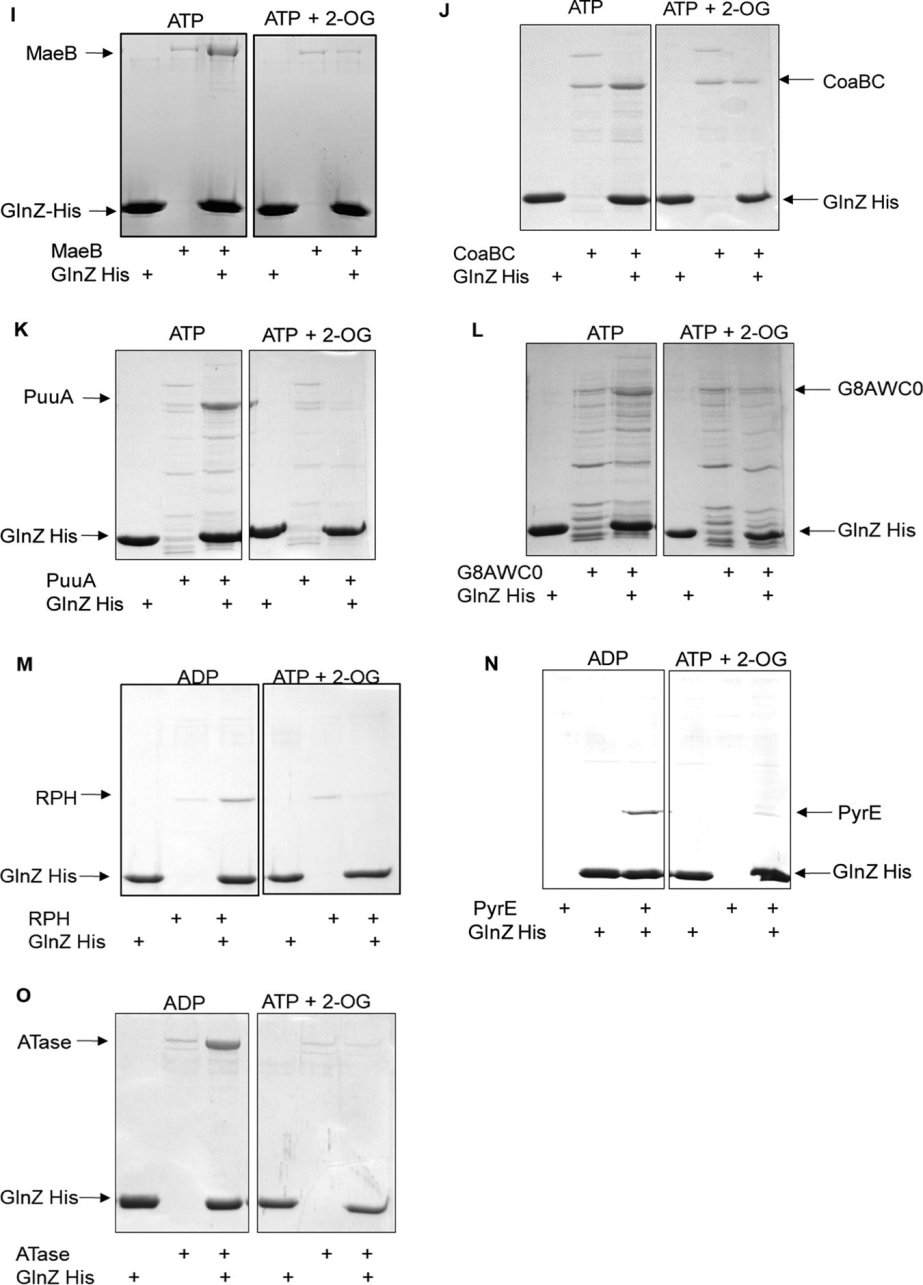
Complex formation between GlnZ and new targets assessed by pulldown. Interactions between purified His-GlnZ and target proteins were assessed by pulldown in the presence of 5 mM MgCl_2_ and the indicated effectors (1 mM ADP, 1 mM ATP, and 1.5 mM 2-OG). (A) TrkA (G8AQ29); (B) DGC, putative diguanylate cyclase (G8AME0); (C) Rho (G8AG60); (D) ppGpp synthetase (G8APW7); (E) putative RNase J (G8AKW1); (F) NAGK (G8AI64); (G) NadE1 (G8ASI0); (H) putative c-di-GMP phosphodiesterase (G8ATR8); (I) MaeB, NADP-dependent malic enzyme (G8AFB3); (J) CoaBC (G8AF97); (K) PuuA (G8ALX8); (L) putative signal transduction histidine kinase (G8AWC0); (M) RPH. RNase PH (G8AGG4); (N) PyrE, orotate phosphoribosyltransferase (G8AJG7); (O) ATase, glutamine synthetase adenylyltransferase (UniProt accession no. A7Y9V0). Twenty micrograms of His-GlnZ was immobilized into magnetic Ni^2+^ beads and mixed with 20 μg of partially purified protein (A, F, G, I, M, N, and O) or cell extracts overexpressing the indicated recombinant protein (B, C, D, E, H, J, K, and L). Proteins were eluted from the Ni^2+^ beads and analyzed by SDS-PAGE. The gels were stained with Coomassie blue. Controls were performed using His-GlnZ or target protein only. Enrichment of the band corresponding to the target protein in the presence of His-GlnZ are indicative of direct complex formation. The specificity of the protein complexes was further validated by their sensitivity in the presence of 2-OG.

Out of the 37 candidate GlnZ-binding proteins, 23 were cloned into expression vectors, and 21 could be expressed in the soluble fraction using E. coli BL21 λ(DE3) as host. From these soluble recombinant proteins, eight were purified to homogeneity and complex formation with His-tagged GlnZ (His-GlnZ) was studied by pulldown using Ni^2+^ beads. The other 13 proteins were tested for interaction with His-GlnZ using the soluble protein fraction of whole E. coli BL21 λ(DE3) cell extract after the induction of the potential GlnZ target recombinant protein. Of the 21 examined proteins, 15 were confirmed as PII interactors by pulldown assays as judged by visual enrichment of the band corresponding to the target protein in the presence of GlnZ in comparison to the controls (Fig. 1 and Table 1). For all complexes confirmed by pulldown, the presence of 2-OG reduced the degree of target protein copurification with GlnZ (Fig. 1), thus providing additional specificity of the protein complex detected.

### Functional groups of the putative novel GlnZ-binding targets. (i) Proteins related to fatty acid metabolism.

It is well established that PII proteins regulate fatty acid biosynthesis from *Bacteria* to plants by direct interaction with BCCP (30–32). Indeed, BCCP was detected among the GlnZ targets in our assays (Table 1). The BCCP protein is a component of the acetyl-CoA carboxylase enzyme (ACC). In most *Bacteria*, including E. coli and *A. brasilense*, ACC is formed by four different subunits, BCCP, BC, CTα, and CTβ. In eukaryotes, including humans, the ACC subunits are encoded by a single polypeptide. Whereas in *Actinobacteria* such as Mycobacterium tuberculosis, the ACC enzymes are formed by two polypeptides, namely, AccA (fused BCCP and BC) and AccD (fused CTα and CTβ) (33). Two subunits of an enzyme annotated as acetyl/propionyl-CoA carboxylase, namely, AccA and AccD, which resembles the organization of ACC from *Actinobacteria* (G8ATT3 and G8ATT5) were detected in our GlnZ-ligand fishing assays (Table 1).

These data suggest that PII proteins may have the principal ability to interact with other proteins containing a BCCP domain in addition to the archetypical BCCP present in *Bacteria* and chloroplasts encoded as a separate polypeptide. Indeed, previous PII-ligand fishing assays in Arabidopsis thaliana indicated that multiple isoforms of BCCP are able to interact with PII in the plant chloroplast (30).

In addition to BCCP, other proteins related to fatty acid metabolism have been detected as potential GlnZ targets (Table 1) including FabG, the enzyme responsible for the first step of fatty acid elongation cycle. The direct interaction between FabG and GlnZ was not confirmed by pulldown (see Fig. S1 in the supplemental material). One possibility is that FabG may have been fished indirectly through complex formation with other direct PII targets such as ACC.

10.1128/mSystems.00817-20.5 FIG S1 Negative complex formation between GlnZ and putative new targets. Interaction between His-GlnZ and putative target proteins (A to E) or His-RpoN and GlnZ (F) was assessed by pulldown in the presence of 5 mM MgCl_2_ and the indicated effectors (1 mM ATP, 1 mM ADP, or 1 mM ATP + 1.5 mM 2-OG). (A) EIIA^Ntr^, PTS nitrogen regulatory IIA protein (G8AZB5); (B) ZapA, putative uncharacterized protein (cell division protein ZapA) (G8AM13); (C) short-chain dehydrogenase/reductase (3-oxoacyl-[acyl-carrier protein] reductase (G8AWA1); (D) SpeE, spermidine synthase (G8AGC5); (E) 3-oxoacyl-(acyl carrier protein) reductase G8ASZ7; (F) RpoN, RNA polymerase sigma-54 factor (G8AT20). Twenty micrograms of His-tagged GlnZ (A to E) or RpoN (F) was immobilized into magnetic Ni^2+^ beads and mixed with 20 μg of purified untagged protein (A and F) or 100 μl of extract containing overexpressed protein of interest (B to E). Proteins eluted from the Ni^2+^ beads were analyzed by SDS-PAGE, and the gels were Coomassie blue stained. Download FIG S1, PDF file, 0.1 MB. Copyright © 2020 Gerhardt et al. 2020 Gerhardt et al. This content is distributed under the terms of the Creative Commons Attribution 4.0 International license.

### (ii) Proteins related to nitrogen metabolism.

The primary function of PII proteins is to regulate nitrogen metabolism. As expected, several proteins related to nitrogen pathways have been detected in our GlnZ-ligand fishing assays (Table 1), including NAGK and ATase, two enzymes that have been extensively studied as PII targets in other organisms (7, 29). Proteins related to nucleotide biosynthesis (PyrE and PurC) and degradation (Ade) along with those involved in polyamine metabolism (PuuA and SpeE) were also identified as potential GlnZ-binding targets (Table 1). Direct protein-protein interaction with GlnZ was confirmed for NAGK, ATase, PuuA, and PyrE as assessed by pulldown (Fig. 1F, O, K, and N).

The mechanism of regulation of glutamine synthetase (GS) adenylylation in *A. brasilense* remained elusive, as PII mutant strains did not affect GS adenylylation *in vivo* (19). The identification of a GlnZ-ATase protein complex suggests that *A. brasilense* may possess a canonical control of GS adenylylation via PII-ATase complex such as that described in E. coli. A better comprehension of the regulatory properties of the PII-ATase complex in *A. brasilense* is key to genetically engineer ammonium-excreting strains with improved biofertilizer traits.

The interaction between PII and NAGK was thought to be restricted to oxygenic phototrophs and evolved early in the cyanobacterial lineage (4). However, recent data indicated that this interaction is also conserved in Gram-positive Corynebacterium glutamicum (34). The finding that PII also interacts with NAGK in the Gram-negative alphaproteobacterium *A. brasilense* (Fig. 1F) supports that the PII-NAGK complex may be much more widespread than originally thought.

The NAGK activity is required for l-arginine biosynthesis. This amino acid is an important precursor for polyamine biosynthesis. Further enzymes involved in polyamine metabolism were recovered as potential novel GlnZ-binding targets (Table 1), such as spermidine synthase (Spe), which converts putrescine to spermidine and gamma-Glu-putrescine synthase (PuuA), involved in putrescine degradation. Complex formation between GlnZ and PuuA could be confirmed using pulldown (Fig. 1K). PuuA belongs to the glutamine synthetase family and shares significant homology with GS. Interestingly, in some *Archaea*, PII proteins regulate GS activity by direct protein-protein interaction (35, 36). Hence, there may be a conserved structural element within the glutamine synthetase family which acts as a docking site for PII proteins.

### (iii) Proteins involved in coenzyme and secondary messenger metabolism.

Two isoforms of the glutamine-dependent NAD synthetase (NadE1 and NadE2) and the enzyme involved in coenzyme A biosynthesis (CoaBC) were identified as potential GlnZ targets (Table 1). The NadE2 enzyme has been studied as a GlnZ target in *A. brasilense* previously (26). Upon an ammonium shock, GlnZ interacts with NadE2 to relieve the NAD^+^ negative-feedback inhibition over the NadE2 enzyme. In the current study, we also identified and validated the interaction between GlnZ and the second glutamine-dependent NAD synthetase isoform NadE1 (Fig. 1G), thus corroborating our previous bioinformatic predictions based on the conservation of PII-NadE gene order. Interestingly, the third NadE isoform present in *A. brasilense*, which does not contain the N-terminal glutaminase domain, was not fished by GlnZ despite being detected by our proteomic analysis in the cell extracts. Hence, we speculate that GlnZ interacts with the glutaminase domain of NadE paralogues. The interaction between CoaBC and GlnZ was confirmed by pulldown (Fig. 1J) though this particular interaction seems to be weak given the small enrichment (but visible and 2-OG responsive) of CoaBC in the presence of His-GlnZ in comparison to the control (Fig. 1J).

Surprisingly, two enzymes involved in the metabolism of the second messenger c-di-GMP were identified as potential GlnZ targets. A putative diguanylate cyclase DGC (G8AME0) and a putative diguanylate cyclase/phosphodiesterase PDE with PAS/PAC domain (G8ATR8). For both enzymes, the interaction was confirmed by pulldown assays and was responsive to the 2-OG levels (Fig. 1B and H). The fact that GlnZ showed a stable interaction with enzymes catalyzing opposing reactions strongly suggests that GlnZ may play a role in the regulation of c-di-GMP levels.

An enzyme annotated as a putative ppGpp synthetase (G8APW7, putative RelA) was also among the list of putative GlnZ targets. This interaction was robust and 2-OG sensitive as judged by pulldown analysis (Fig. 1D). In response to amino acid starvation, RelA interacts with stalled ribosomes activating the synthesis of guanosine tetraphosphate (p)ppGpp. These alarmones globally regulate transcription and translation in a process known as the stringent response, lowering stable RNA synthesis while increasing the transcription of genes involved in amino acid biosynthesis (37). Given the key role of PII proteins in nitrogen sensing, we speculate that GlnZ may affect ppGpp levels and stringent response by direct interaction with RelA. Interestingly, a functional relation between PII proteins and RelA has previously been established in E. coli, where PII regulates the expression of *relA* through the NtrBC nitrogen two-component system (38). Our data suggest that PII may regulate RelA function at the enzyme activity level.

### (iv) Proteins involved in RNA catabolism and transcription.

Four putative enzymes involved in RNA catabolism were identified as potential GlnZ targets. Two of them, namely, the putative ribonucleases J (G8AKW1) and H (Rph), were confirmed to directly interact with GlnZ in a 2-OG-regulated manner by pulldown (Fig. 1E and M). The other RNases G and R were not tested; therefore, it remains to be determined whether RNases G and R directly interact with GlnZ or whether they assemble indirectly via an RNase degradosome, which occurs in other *Bacteria* (39). In any case, our data suggest that GlnZ may be implicated in the regulation of RNA degradation.

Four proteins involved in transcription were among the putative GlnZ-binding partners. These proteins included RpoN, NtrX, Rho factor, and a putative signal transduction histidine kinase (G8AWC0). The interaction between GlnZ and both Rho and G8AWC0 was confirmed by pulldown, and these protein complexes were responsive to 2-OG levels (Fig. 1C and L). The RpoN interaction was not confirmed by pulldown (see Fig. S1F in the supplemental material), while the interaction with NtrX was not further investigated.

### (v) Proteins belonging to other functional groups.

Other proteins that were confirmed to interact with GlnZ by pulldown were TrkA and MaeB1 (Fig. 1A and I). The MaeB1 enzyme is similar to malic enzymes containing a nonfunctional C-terminal phosphotransacetylase domain (40). It is believed that MaeB enzymes act in gluconeogenesis by converting l-malate to pyruvate while reducing NADP to NADPH (41). The *A. brasilense* MaeB1 activity is regulated by the ratio of acetyl-CoA to CoA-SH, thereby controlling the fate of carbon distribution at the phosphoenolpyruvate/pyruvate/oxaloacetate metabolic node (42).

TrkA interacts with the integral membrane TrkH potassium channel to regulate its activity. In E. coli, TrkA function is regulated by nitrogen availability through direct interaction with nitrogen regulatory IIA protein (EIIA^Ntr^) (43). The E. coli TrkA-EIIA^Ntr^ complex responds to l-glutamine and 2-OG levels, as these metabolites regulate EIIA^Ntr^ phosphorylation (44). Interestingly, a putative EIIA^Ntr^ was also detected in our GlnZ ligand fishing assay (Table 1). However, the GlnZ-EIIA^Ntr^ complex could not be confirmed by pulldown (Fig. S1A). One possibility is that GlnZ and EIIA^Ntr^ may interact indirectly through the formation of a GlnZ-TrkA-EIIA^Ntr^ ternary complex. Alternatively, the interaction may occur with phosphorylated EIIA^Ntr^ which was not investigated here.

Several studies indicate a link between ammonium and potassium metabolism in prokaryotes. Given the similarities in ionic radii and hydration shells between NH_4_^+^ and K^+^, membrane transporters for these ions allow significant amounts of the similar ion to permeate (11). One possibility is that the purpose of the interaction between TrkA and GlnZ is to regulate the transport function in response to the ammonium levels in analogy to the AmtB-PII complex (45). Furthermore, the interaction between PII and Trk may function to tune K^+^ transport to l-glutamate levels, since K^+^ acts as the major counter ion to l-glutamate *in vivo*. Both l-glutamate and K^+^ pools increase when nitrogen-limited E. coli is supplied with ammonium (46); it is tempting to speculate that both responses are orchestrated by PII proteins.

### Untargeted metabolome analysis of the *A. brasilense glnB glnZ* double mutant strain.

Given the vast range of proteins identified as potential novel targets of the PII protein GlnZ in *A. brasilense*, we sought that a PII-minus strain would present an altered metabolome. Hence, LC-MS was used to compare the metabolome of wild-type *A. brasilense* versus a *glnB glnZ* double mutant strain. Data were obtained from cells collected during nitrogen starvation (−N) and 5 min after the addition of 1 mM NH_4_Cl (+N). Upon the addition of ammonium to nitrogen-starved cells, we expect that PII proteins became deuridylylated and without bound 2-OG, thereby resembling the conditions used in our GlnZ ligand fishing assays. The *glnB glnZ* double mutant strain was used in metabolic analysis, since literature data indicate that the multiple PII paralogues may have overlapping functions (1). Hence, metabolic defects could be masked in a *glnZ* single mutant strain due to redundant functions of the *glnB* paralogue.

The biological triplicate LC/MS run from cells cultured in −N and +N were compared using pair and multigroup XCMS analysis (47). Nonmetric multidimensional scaling (NMDS) indicated a clear separation of the data according to strain at dimension 1 (Fig. S2). A total of 19 metabolic pathways were disturbed in the *A. brasilense* 2812 *glnB glnZ* double mutant strain at −N in pairwise comparison to the FP2 wild-type strain (*P* < 0.05) (Table S2A). Five minutes after the addition of ammonium (+N), the number of disturbed pathways in the 2812 *glnB glnZ* double mutant strain was 82 (Table S2B). The increasing number of disrupted pathways in response to an ammonium shock in the PII mutant reinforces the role of PII proteins in metabolic regulation in response to nitrogen availability.

10.1128/mSystems.00817-20.3 TABLE S2 Metabolic effects in *glnB glnZ* double mutant strain. (A) Metabolic pathways altered in the *glnB glnZ* double mutant strain in −N condition in pairwise comparison to the FP2 wild-type strain (*P* < 0.05). (B) Metabolic pathways altered in the *glnB glnZ* double mutant strain in +N condition in pairwise comparison to the FP2 wild-type strain (*P* < 0.05). (Column 1) Number of significantly dysregulated metabolites found in the pathway. (Column 2) Total number of putative metabolites found in the pathway using the entire LC-MS analysis results. Download Table S2, XLSX file, 0.01 MB. Copyright © 2020 Gerhardt et al. 2020 Gerhardt et al. This content is distributed under the terms of the Creative Commons Attribution 4.0 International license.

10.1128/mSystems.00817-20.6 FIG S2 Nonmetric multidimensional scaling (NMDS) of the raw LC-MS metabolome data. Triplicate samples of FP2 –N (light blue), FP2 +N (dark blue), *glnBglnZ* minus strain 2812 –N (green), and *glnBglnZ* minus strain 2812 +N (red). Download FIG S2, PDF file, 0.1 MB. Copyright © 2020 Gerhardt et al. 2020 Gerhardt et al. This content is distributed under the terms of the Creative Commons Attribution 4.0 International license.

Most of the disrupted pathways in the PII-deficient strain compared to the wild-type strain at −N conditions were related to nucleotide, glycolysis, gluconeogenesis, TCA, and trehalose and histidine metabolism. After the ammonium shock (+N), additional nucleotide metabolic pathways were disturbed in the mutant along other pathways, including those involved in the metabolism of amino acids, NAD^+^, and phospho/amino sugar (Table S2). These data reinforce the role of PII proteins in the regulation of amino acid and NAD^+^ in response to ammonium availability. Activity network analysis connecting all disrupted metabolites is depicted in Fig. S3. These global metabolic defects may explain the impaired growth previously reported in the 2812 *glnB glnZ* double mutant strain (19).

10.1128/mSystems.00817-20.7 FIG S3 Activity network metabolome analysis. XCMS graphical representation of the metabolites from the pathways which have been identified in the data set based on their pathway connections. The *P* value of the features and their adduct combination are taken and mapped onto the E. coli Biosource pathway. The color of the nodes represents the change in a pairwise analysis (red [upregulated] and purple [down regulated in the wild-type strain in comparison to the *glnBglnZ* minus strain]). (A) Data from pairwise analysis in the –N condition. (B) Data from pairwise analysis in the +N condition. Download FIG S3, PDF file, 0.1 MB. Copyright © 2020 Gerhardt et al. 2020 Gerhardt et al. This content is distributed under the terms of the Creative Commons Attribution 4.0 International license.

**FIG 2:**
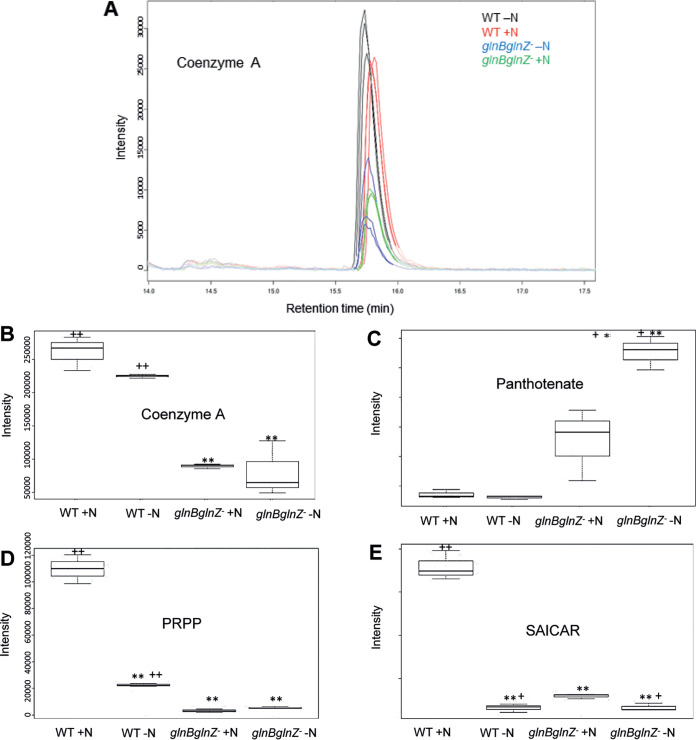
Quantitative data of selected metabolites. (A) Extracted ion chromatogram (EIC) for coenzyme A (M-H, 766.0997 to 766.1118 *m/z*) in the biological triplicates. Box plot analysis of EIC data at 5 ppm for coenzyme A (B), panthothenate (C), PRPP (D), SAICAR (E). *A. brasilense* FP2 wild-type strain (WT) and *A. brasilense* 2812 (*glnBglnZ*) under nitrogen limitation (–N) and 5 min after the addition of ammonium (+N) are shown. Statistical significance by *t* test: *, *P* < 0.05; **, *P* < 0.01 [an asterisk(s) indicates that the statistical analysis was comparing the value to the value for WT +N]; +, *P* > 0.05; ++, *P* < 0.01 [a plus sign(s) indicates that the statistical analysis was comparing the value to the value for *glnBglnZ* +N].

We manually investigated metabolites that act as the substrates or products of the enzymes listed as potential novel GlnZ-binding targets. Efforts were concentrated in compounds identified as [M-H]-, with less than 5 ppm mass deviation and expected elution time based on the literature (48). We observed that the level of coenzyme A (CoA) was reduced in the PII-deficient strain in both −N and +N conditions (Fig. 2A and B). On the other hand, pantothenate, which is a precursor of CoA biosynthesis, showed the opposite trend (Fig. 2C). These data point to dysregulated CoA biosynthesis in the PII mutant strain, which could be explained by the fact that CoaBC, an enzyme involved in CoA biosynthesis, was identified as a novel GlnZ target (Table 1). Alternatively, dysregulated CoA levels may be caused by the fact that PII regulates enzymes that use acetyl-CoA such as acetyl-CoA carboxylase. Unfortunately, we could not detect esterified forms of CoA in our LC-MS data.

In the wild-type cells, the levels of the nucleotide precursors PRPP (phosphoribosyl pyrophosphate) and SAICAR (phosphoribosylaminoimidazolesuccinocarboxamide) augmented in response to the ammonium shock (Fig. 2D and E). Conversely, in the PII-deficient mutant, PRPP and SAICAR were lower and less responsive to nitrogen (Fig. 2D and E). The enzymes PyrE and PurC, which were identified in the GlnZ ligand fishing assays, have PRPP and SAICAR as the substrate and product, respectively. Hence, these data provide additional support for a role of PII proteins in the regulation of PyrE and PurC.

### The GlnZ protein regulate c-di-GMP levels *in vivo* and cell motility.

Among the novel GlnZ targets identified and validated by pulldown, there were two enzymes involved in c-di-GMP metabolism with opposing activities, a putative diguanylate cyclase DGC, G8AME0, and a putative diguanylate cyclase/phosphodiesterase PDE, G8ATR8 (Table 1). We thought that the interaction with GlnZ would result in the activation/deactivation of the opposing enzyme activities in order to avoid a futile cycle of c-di-GMP synthesis and degradation. If that hypothesis is correct, dysregulated c-di-GMP levels should be observed in a *glnZ* knockout strain.

**FIG 3:**
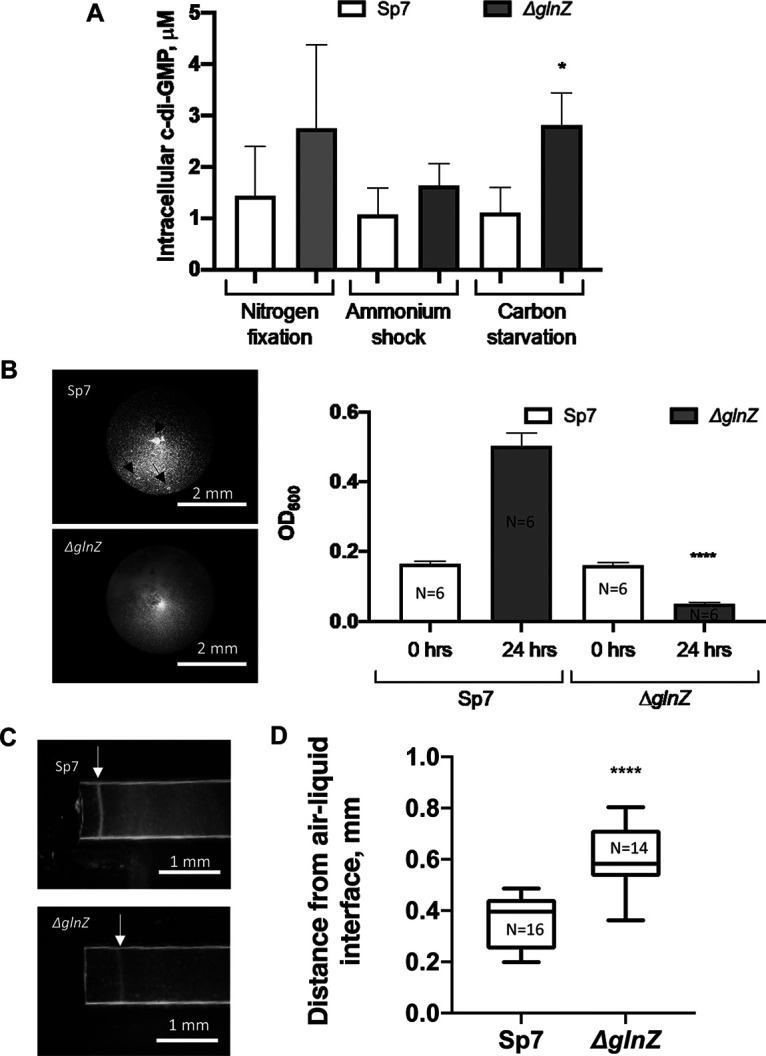
The GlnZ protein is involved in the control of c-di-GMP levels and related cellular behaviors. (A) Intracellular c-di-GMP levels in *A. brasilense* wild-type strain (Sp7) and *glnZ*-minus strain (Δ*glnZ*) under indicated metabolic conditions. Statistical significance: *, *P < *0.05. (B) Flocculation, as determined by the OD of the supernatant of cell suspensions in which cells were induced to flocculate for 24 h. Statistical significance: ****, *P < *0.0001. N, number of replicates. The picture shows flocculated cells indicated by black arrows. (C) Capillary aerotaxis assays. The white arrow indicates the formation of a biomass band of motile cells 120 s after the exposure to ambient air. The band was stable for over 15 min under these conditions. (D) Distance of the aerotaxis biomass band from the end of the capillary tube. Statistical significance: ****, *P* < 0.0001. N, number of replicates.

The levels of c-di-GMP were determined *in vivo* in *A. brasilense* using target LC-MS/MS analysis. We determined c-di-GMP levels in cells collected during nitrogen starvation (−N) and 5 min after the addition of NH_4_Cl (+N) or with a low concentration of the carbon source and high ammonium (carbon starvation) (Fig. 3A). The *glnZ*-minus strain showed a trend of increased c-di-GMP under all conditions although statistical significant increase was detected only under carbon starvation (Fig. 3A). This correlates well with the results of our pulldown assays which suggest that GlnZ interacts with c-di-GMP-related enzymes only under low 2-OG levels which are likely to occur *in vivo* exactly under carbon starvation (under low carbon and high nitrogen). These data support that GlnZ regulates c-di-GMP homeostasis *in vivo*.

It is well established in different bacteria that increased c-di-GMP levels are associated with the transition of bacteria from motile and planktonic to sessile lifestyles (49). Consistent with this hypothesis, the *glnZ*-minus strain flocculated more than the wild type (Fig. 3B). The levels of c-di-GMP also modulate aerotaxis in *A. brasilense* (50). Hence, we compared the aerotaxis behavior between the wild type and the *glnZ* mutant strain. While the *glnZ* mutant strain was motile, it showed impaired aerotaxis, localizing at a different zone, relative to the wild-type strain, in aerotaxis capillary assays (Fig. 3C and D).

## DISCUSSION

The aim of this work was to provide a comprehensive analysis of the GlnZ interaction network in *A. brasilense*. The use of GlnZ affinity columns coupled to mass spectrometry has been used previously by our group to successfully identify novel targets of PII in *A. brasilense* such as BCCP and NadE2 (28). By systematically analyzing the proteins retrieved from multiple GlnZ ligand fishing assays, a list of 37 proteins that are likely to interact with GlnZ was generated. Among the potential novel GlnZ-binding targets were proteins related to nitrogen and fatty acid metabolism, signaling, coenzyme synthesis, RNA catabolism, and transcription (Table 1). These data suggest that PII proteins may play a much broader role in metabolic regulation than previously envisaged by the literature.

We cannot exclude the possibility that some of the proteins identified as GlnZ-binding targets may be false-positive results. Moreover, some of the PII protein interactions identified may occur indirectly which is likely to be the case of the AccD (G8ATT5). In order to provide additional evidence for direct protein-protein interaction with GlnZ, candidate proteins were expressed and subjected to *in vitro* pulldown assays with GlnZ. These analyses, along with previously published data confirmed that GlnZ is able to directly interact with 17 of these proteins (Table 1). In all cases, complex formation was confirmed by pulldown assays and the protein complexes were sensitive to 2-OG, thereby further validating the specificity of the assay (Fig. 1).

Untargeted metabolome analysis revealed altered levels of coenzyme A, pantothenate, PRPP, and SAICAR in a PII-deficient strain (Fig. 2). Given that enzymes involved with the use/production of these metabolites were identified as potential targets of GlnZ, the altered accumulation of these metabolites in a PII-minus strain provides additional evidence for the implication of PII in the regulation of these enzymes. The central metabolite PRPP acts as a major carbon skeleton for the biosynthesis of purine and pyrimidine nucleotides, the amino acids histidine and tryptophan, and the cofactors NAD and NADP (51). The metabolome analysis revealed that PRPP levels increase in *A. brasilense* wild-type strain in response to ammonium availability (Fig. 2D). This metabolic response may be important to provide enough carbon skeleton for nucleotide biosynthesis when nitrogen becomes available. On the other hand, PRPP levels remained low in the PII-deficient mutant despite nitrogen availability (Fig. 2D), supporting a role of PII in the regulation of PRPP levels. A similar profile was observed for the metabolite SAICAR (Fig. 2E), which takes part in the purine biosynthesis pathway (52). The metabolic defects may explain the severe growth defects previously reported in the *glnB glnZ* double mutant (19). Other groups of metabolites that were affected by the absence of PII signaling (see Table S2 and Fig. S3 in the supplemental material) include those involved in NAD metabolism, reinforcing the previously described role of PII on the regulation of NAD biosynthesis.

An important outcome of the current study was the finding that proteins from diverse functional groups were among the validated GlnZ-binding targets (Table 1). We surmise that they could possess recurring structural elements that would act as the docking sites for GlnZ. Protein domain/motif analyses were performed with the pulldown validated GlnZ targets. The recurring protein domains/motifs were biotinyl/lipoyl-binding, GGDEF, CN-hydrolase, PAS, NAD(P)-binding, and coiled-coil, suggesting that these domains could be candidates for docking of GlnZ. Interestingly, the GAF domain of the NifA protein has been already described as the docking site for PII proteins in *A. brasilense* (53).

A recent study used PII ligand fishing assays to elucidate the PII protein interaction network in *Synechocystis* sp., revealing that PII controls a range of transporter systems via direct protein-protein interaction (54). Strikingly, the only common protein identified as a PII target between that study and in the current study was NAGK. The distinct PII interaction network detected in *Synechocyctis* sp. and *A. brasilense* may reflect different metabolism operating in these organisms, i.e., phototrophic versus heterotrophic metabolism. Alternatively, it may be caused by the lack of the PII effectors (ATP, ADP, and 2-OG) during the ligand fishing assays performed in *Synechocyctis* sp. We are currently exploring the PII interaction network in other organisms under similar experimental conditions to establish whether there are other across domain conserved PII-target complexes such as the PII-NAGK complex.

It is well established that the ancient multitasking PII proteins orchestrate metabolic regulation in *Bacteria*, *Archaea*, and plants. In this study, we provide evidence that the *A. brasilense* PII protein GlnZ regulates a broad range of metabolic pathways such as those involved with nitrogen and fatty acid metabolism, signaling, coenzyme synthesis, RNA catabolism, and transcription. Untargeted metabolome analysis confirmed dysregulated levels of important cellular metabolites in the absence of PII. Given the ancestry and the extraordinary ability of PII proteins to sense and integrate the levels of key metabolic signals such as ATP, ADP, 2-OG, and l-glutamine, it would not be surprising if PII would act as a master metabolic regulator. Even though our data show that PII interacts with various key metabolic proteins, further biochemical studies will be required to elucidate whether and how the PII proteins affect the activity of the identified PII target protein through complex formation. In addition to direct regulation of the target protein(s) activity, it is possible that regulation occurs by controlling cellular localization of protein(s), as reported for DraG in *A. brasilense* (55, 56).

Among the novel GlnZ targets identified and validated by pulldown, there were two enzymes involved in c-di-GMP metabolism with opposing activities (Table 1). *In vivo* determination of c-di-GMP revealed altered accumulation of c-di-GMP in a *glnZ-*deficient mutant, suggesting altered regulation of c-di-GMP-producing/degrading enzyme in the absence of GlnZ (Fig. 3A). Such a hypothesis is supported by a phenotypic analysis, which showed that the *glnZ*-minus strain has increased flocculation (Fig. 3B) and altered aerotaxis behavior (Fig. 3C and D). Both these phenotypes are well established to be linked to intracellular c-di-GMP levels (49, 57). Further biochemical and genetic analysis will be required to determine the exact mechanism of how GlnZ controls c-di-GMP signaling and these cellular behaviors. It is worth mentioning that while most of the detected nucleotides were either slightly downregulated or unaffected in the PII mutant strain, the level of CMP was higher in the PII mutant (2.2× and 6.7× in the −N and +N conditions, respectively) (Fig. S4). Given that CMP is the final product of c-di-GMP degradation, the altered levels of c-di-GMP and CMP may be caused by a dysregulated cycle of c-di-GMP synthesis/degradation.

10.1128/mSystems.00817-20.8 FIG S4 Quantitative data of CMP. Box plot analysis of EIC data at 5 ppm for CMP. WT, *A. brasilense* wild-type strain; *glnBglnZ*, *A. brasilense* 2812; –N, nitrogen limitation; +N, 5 min after the addition of ammonium. Statistical significance by *t* test: *, *P < *0.05; ** *P < *0.01 (* statistical analysis comparing to WT +N); +, *P < *0.05; ++ *P < *0.01 (+ statistical analysis comparing to *glnBglnZ* +N). Download FIG S4, PDF file, 0.04 MB. Copyright © 2020 Gerhardt et al. 2020 Gerhardt et al. This content is distributed under the terms of the Creative Commons Attribution 4.0 International license.

These data lead us to propose that GlnZ acts to regulate the opposing enzyme activities of c-di-GMP synthesis/degradation supporting a novel role of PII proteins in c-di-GMP signaling. The GlnZ protein could be used as a processing unit to sense key nutrients (2-OG and l-glutamine) and energy (ATP/ADP ratio) to regulate c-di-GMP levels, thereby allowing the cells to make the proper decision, i.e., to be planktonic or sessile, in response to environmental signals. Whether regulation of c-di-GMP levels by PII proteins operate in other prokaryotes besides *A. brasilense* remains to be determined. However, an inspection at the Pfam database (58) indicated a guanylate cyclase domain fused to PII in sequences from the *Archaea Nitrosotalea* (pfam A0A2H1FDM3_9ARCH) suggesting that PII may regulate c-di-GMP in other taxa. Interestingly, recent data in the plant-pathogen bacterium Dickeya dadantii point to an association between the levels of TCA intermediates and c-di-GMP (59). It is tempting to speculate that such connection involves the PII proteins via 2-OG sensing.

The ubiquitous distribution suggests that PII evolved in a deep branching common ancestor and was kept conserved during the evolution of *Archaea*, *Bacteria*, and chloroplasts. The ancient appearance of PII supports that it coevolved with most of the metabolic pathways we know today. It would not be surprising if nature had capitalized the sensory properties of PII to regulate a vast range of key enzymes and proteins according to their metabolic needs, as supported by our data. Further biochemical analysis of each of the novel PII complexes and decoding the PII interaction network in other organisms are under way to provide additional details in the function of these extraordinary sensory proteins.

## MATERIALS AND METHODS

### Bacterial strains and plasmids.

The bacterial strains, plasmids, and primers used are listed in Table S3 in the supplemental material. General cloning methods are described in Text S1 in the supplemental material.

10.1128/mSystems.00817-20.1 TEXT S1 Detailed description of Materials and Methods. Download Text S1, DOCX file, 0.04 MB. Copyright © 2020 Gerhardt et al. 2020 Gerhardt et al. This content is distributed under the terms of the Creative Commons Attribution 4.0 International license.

10.1128/mSystems.00817-20.4 TABLE S3 Synthetic genes, primers, strains, and plasmids. Download Table S3, DOCX file, 0.1 MB. Copyright © 2020 Gerhardt et al. 2020 Gerhardt et al. This content is distributed under the terms of the Creative Commons Attribution 4.0 International license.

### His-tagged GlnZ Ni^2+^ affinity column interaction.

Three hundred milliliters of E. coli BL21(DE3) carrying the pMAS3 plasmid, expressing the *A. brasilense* HisGlnZ protein (60) was cultivated in LB at 37°C until an optical density of 600 nm (OD_600_) of 0.5 was reached when 0.5 mM isopropyl-β-d-thiogalactopyranoside (IPTG) was added, following 4-h incubation. Cells were collected by centrifugation and resuspended in buffer 1 (50 mM Tris-HCl [pH 8], 100 mM KCl, and 20 mM Imidazole). Cells were disrupted by sonication, and the soluble fraction obtained after centrifugation (30,000 × *g* at 4°C for 30 min) was loaded onto a 1-ml Hi-trap chelating column (GE Healthcare) or onto a 1-ml Protino Ni-IDA column (Macherey-Nagel). The column was washed with 15 ml of buffer 1 containing 50 mM imidazole to remove unspecific bound proteins. The column was equilibrated with 5 ml of buffer 2 (buffer 1 containing 5 mM MgCl_2_, 1 mM ATP or ADP as indicated in each experiment, and 20 mM imidazole) before the addition of the *A. brasilense* protein extract.

To prepare the A. brasilense protein extract, 400 ml of the *A. brasilense* 2812 strain was cultivated on NFbHP medium (66) to an OD_600_ of 2. Cells were collected by centrifugation, resuspended in 20 ml of buffer 2 (containing 1 mM ATP or ADP as indicated in each experiment), and disrupted by sonication. The soluble fraction (after centrifugation at 30,000 × *g*, 4°C for 30 min) was divided into two aliquots. One was loaded onto the HisGlnZ charged Ni^2+^ column, and the other aliquot was loaded onto a control Ni^2+^ column (without preloaded HisGlnZ). Both columns were washed with 20 ml of buffer 2. The putative GlnZ-interacting proteins were specifically eluted by passing 3 ml of 2-OG elution buffer (buffer 1 containing 5 mM MgCl_2_, 1 mM ATP, and 1.5 mM 2-OG). The eluted fractions were analyzed by SDS-PAGE, MALDI-TOF (details in Text S1), and label-free quantitative LC-MS/MS.

### FLAG-tagged GlnZ affinity column interaction.

Two separate aliquots of 300 μl of anti-FLAG M2 magnetic beads (Sigma) were washed twice with 500 μl of buffer A (50 mM Tris-HCl [pH 8], 100 mM KCl, 1 mM ATP, and 5 mM MgCl_2_). One aliquot was used as a negative control, while the other was loaded with cell extracts containing overexpressed *A. brasilense* 3×-FLAG GlnZ. After 15 min at room temperature, beads were washed three times with buffer A. Both control and 3×-FLAG GlnZ-loaded beads were incubated with 1.5 ml of *A. brasilense* 2812 protein extract (prepared as described in the previous section, in buffer A). After 30 min at room temperature, beads were washed four times with 500 μl of buffer A. Elution was achieved by two consecutive 2× washes with 100 μl of buffer A containing 1.5 mM 2-OG. The eluted fractions of control and FLAG-tagged GlnZ affinity column were analyzed by label-free quantitative LC-MS/MS and MALDI-TOF analysis (details in Text S1).

### Label-free quantitative LC-MS/MS analysis.

A total of six independent assays were considered for the analyses. In four assays, proteins were allowed to interact with GlnZ in the presence of ATP and were eluted in the presence of ATP plus 2-OG (namely, ATP1 to ATP4). In two assays, proteins were allowed to interact with GlnZ in the presence of ADP and were eluted in the presence of ATP plus 2-OG (namely, ADP1 and ADP2).

Assays ATP1 and ATP2 were performed using Ni^2+^ HiTrap chelating columns (GE Healthcare), assay ATP3 was performed using Protino Ni-IDA column (Macherey-Nagel), and assay ATP4 was performed using anti-FLAG M2 magnetic beads (Sigma-Aldrich). The rationale of using different chromatographic matrices was to discard proteins that could be enriched due to spurious affinity to the matrix. Both ADP1 and ADP2 assays were performed using Ni^2+^ HiTrap chelating columns (GE Healthcare).

Proteins eluted from His-tagged GlnZ Ni^2+^ affinity columns or FLAG-tagged GlnZ affinity columns were analyzed by label-free LC-MS/MS as described previously (61) (details in Text S1).

### Protein complex pulldown assays using nickel magnetic beads.

*In vitro* protein complex formation was assessed using MagneHis beads (Promega) as described previously (22). All reactions were conducted in buffer containing 50 mM Tris-HCl (pH 8), 0.1 M NaCl, 10% glycerol, and 20 mM imidazole in the presence of PII effectors as indicated in each experiment. Details are provided in Text S1.

### Untargeted metabolomic analysis of *A. brasilense*.

The *A. brasilense* wild-type FP2 or 2812 (PII double mutant *glnB glnZ*) strains were cultured in triplicate vials in NFbHP medium containing 20 mM NH_4_Cl at 30°C, 120 rpm until an OD_600_ of 1 was reached. Cells were collected by centrifugation (2,000 × *g*, 10 min, 4°C), resuspended, and maintained for 2 h at 30°C and 120 rpm in NFbHP without fixed nitrogen aerobically in order to obtain nitrogen-starved cells (−N) and induce PII protein expression. Aliquots of the cell cultures (15 ml) were collected before (−N) and 5 min after the addition of NH_4_Cl 1 mM (+N). Cells were processed and analyzed by LC-MS as previously described (62), with modifications (details in Text S1).

The LC-MS data generated from independent triplicate samples were analyzed using XCMS online (47). Feature detection was set as follows: 5 ppm; minimum and maximum peak width, 10 and 30, respectively; signal/noise threshold, 20; *m/z* diff, 0.03. Feature annotation was performed using 10 ppm with an absolute error of 0.02.

To quantify c-di-GMP levels *in vivo*, *A. brasilense* wild-type strain Sp7 and strain 7611 (*glnZ* minus) were cultured in minimal medium containing 20 mM NH_4_Cl and 10 mM l-malate as the carbon source to an OD_600_ of 0.5. Cells were washed and resuspended in media containing 10 mM l-malate without nitrogen to induce nitrogen fixation and GlnZ protein expression. The cultures were split into two 10-ml samples. c-di-GMP was extracted from one 10-ml sample to establish basal c-di-GMP levels (nitrogen fixation condition). The other 10-ml sample was subjected to an ammonium shock (20 mM NH_4_Cl) for 30 min before c-di-GMP extraction. In a third condition, named carbon starvation, cells were incubated for 30 min in media containing 1 mM l-malate and 20 mM NH_4_Cl before c-di-GMP extraction. The 10-ml cell aliquots were collected by centrifugation, pellets were weighed and treated in a manner similar to that described above (see Text S1 for details). Samples were shipped overnight to Michigan State University mass spectrometry and metabolomics facility where c-di-GMP was quantified by LC-MS/MS as described previously (63).

### Phenotypic analysis.

The cell swimming aerotaxis assays were prepared and analyzed as previously described (57) (see Text S1 for details). For flocculation assays, the *A. brasilense* strains were grown in 5 ml of MMAB (67) supplemented with 20 mM NH_4_Cl and 10 mM malate to an OD_600_ of 0.8. Cells were centrifuged and washed with chemotaxis buffer (K_2_HPO_4_ [1.7 g·liter^−1^], KH_2_PO_4_ 1[.36 g·liter^−1^]). Cultures were resuspended to a final OD_600_ of 0.16 in 1 ml of flocculation medium (64) containing 0.5 mM NH_4_Cl and 8 mM malate. Cultures were placed in 24-well plates and grown at room temperature for 24 h, with shaking at 60 rpm. Supernatants of cell suspensions (therefore excluding flocculated cells that fell at the bottom of the wells) were transferred to cuvettes to measure OD, using gel-loading tips (FisherScientific).

### Statistical analysis.

Statistical analysis was performed in GraphPad Prism 7 using the *t* test. Data were considered significant when *P* values were <0.05.

### Data availability.

The mass spectrometry proteomics data have been deposited to the ProteomeXchange Consortium via the PRIDE repository (65), data set identifier PXD018530.

## References

[1] Huergo LF, Chandra G, Merrick M 2013 PII signal transduction proteins: nitrogen regulation and beyond. FEMS Microbiol Rev 37:251–283. doi:10.1111/j.1574-6976.2012.00351.x. 22861350

[2] Selim KA, Ermilova E, Forchhammer K 2020 From Cyanobacteria to Archaeplastida: new evolutionary insights into PII signaling in the plant kingdom. New Phytol 227:722–731. doi:10.1111/nph.16492. 32077495

[3] Shapiro BM 1969 Glutamine synthetase deadenylylating enzyme system from *Escherichia coli*. Resolution into two components, specific nucleotide stimulation, and cofactor requirements. Biochemistry 8:659–670. doi:10.1021/bi00830a030. 4893578

[4] Forchhammer K, Lüddecke J 2016 Sensory properties of the P_II_ signalling protein family. FEBS J 283:425–437. doi:10.1111/febs.13584. 26527104

[5] Forchhammer K, Selim KA 2020 Carbon/nitrogen homeostasis control in cyanobacteria. FEMS Microbiol Rev 44:33–53. doi:10.1093/femsre/fuz025. 31617886 PMC8042125

[6] Xu Y, Cheah E, Carr PD, van Heeswijk WC, Westerhoff HV, Vasudevan SG, Ollis DL 1998 GlnK, a PII-homologue: structure reveals ATP binding site and indicates how the T-loops may be involved in molecular recognition. J Mol Biol 282:149–165. doi:10.1006/jmbi.1998.1979. 9733647

[7] Jiang P, Ninfa AJ 2009 Reconstitution of *Escherichia coli* glutamine synthetase adenylyltransferase from N-terminal and C-terminal fragments of the enzyme. Biochemistry 48:415–423. doi:10.1021/bi801775b. 19105634 PMC2651632

[8] Fokina O, Herrmann C, Forchhammer K 2011 Signal-transduction protein P II from *Synechococcus elongatus* PCC 7942 senses low adenylate energy charge in vitro. Biochem J 440:147–156. doi:10.1042/BJ20110536. 21774788

[9] Gerhardt ECM, Araújo LM, Ribeiro RR, Chubatsu LS, Scarduelli M, Rodrigues TE, Monteiro RA, Pedrosa FO, Souza EM, Huergo LF 2012 Influence of the ADP/ATP ratio, 2-oxoglutarate and divalent ions on *Azospirillum brasilense* PII protein signalling. Microbiology (Reading) 158:1656–1663. doi:10.1099/mic.0.058446-0. 22461486

[10] Da Rocha RA, Weschenfelder TA, De Castilhos F, De Souza EM, Huergo LF, Mitchell DA 2013 Mathematical model of the binding of allosteric effectors to the *Escherichia coli* PII signal transduction protein GlnB. Biochemistry 52:2683–2693. doi:10.1021/bi301659r. 23517273

[11] Huergo LF, Dixon R 2015 The emergence of 2-oxoglutarate as a master regulator metabolite. Microbiol Mol Biol Rev 79:419–435. doi:10.1128/MMBR.00038-15. 26424716 PMC4651028

[12] Truan D, Huergo LF, Chubatsu LS, Merrick M, Li X-D, Winkler FK 2010 A new PII protein structure identifies the 2-oxoglutarate binding site. J Mol Biol 400:531–539. doi:10.1016/j.jmb.2010.05.036. 20493877

[13] Fokina O, Chellamuthu V-R, Forchhammer K, Zeth K 2010 Mechanism of 2-oxoglutarate signaling by the *Synechococcus elongatus* PII signal transduction protein. Proc Natl Acad Sci U S A 107:19760–19765. doi:10.1073/pnas.1007653107. 21041661 PMC2993416

[14] Jiang P, Ninfa AJ 2009 α-Ketoglutarate controls the ability of the *Escherichia coli* PII signal transduction protein to regulate the activities of NRII (NtrB) but does not control the binding of PII to NRII. Biochemistry 48:11514–11521. doi:10.1021/bi901158h. 19877669 PMC2786246

[15] van Heeswijk WC, Westerhoff HV, Boogerd FC 2013 Nitrogen assimilation in *Escherichia coli*: putting molecular data into a systems perspective. Microbiol Mol Biol Rev 77:628–695. doi:10.1128/MMBR.00025-13. 24296575 PMC3973380

[16] Merrick M 2015 Post-translational modification of PII signal transduction proteins. Front Microbiol 5:763. doi:10.3389/fmicb.2014.00763. 25610437 PMC4285133

[17] Chellamuthu V-R, Ermilova E, Lapina T, Lüddecke J, Minaeva E, Herrmann C, Hartmann MD, Forchhammer K 2014 A widespread glutamine-sensing mechanism in the plant kingdom. Cell 159:1188–1199. doi:10.1016/j.cell.2014.10.015. 25416954

[18] Huergo LF, Pedrosa FO, Muller-Santos M, Chubatsu LS, Monteiro RA, Merrick M, Souza EM 2012 P II signal transduction proteins: pivotal players in post-translational control of nitrogenase activity. Microbiology (Reading) 158:176–190. doi:10.1099/mic.0.049783-0. 22210804

[19] De Zamaroczy M 1998 Structural homologues P(II) and P(z) of *Azospirillum brasilense* provide intracellular signalling for selective regulation of various nitrogen-dependent functions. Mol Microbiol 29:449–463. doi:10.1046/j.1365-2958.1998.00938.x. 9720864

[20] Rodrigues TE, Souza VEP, Monteiro RA, Gerhardt ECM, Araújo LM, Chubatsu LS, Souza EM, Pedrosa FO, Huergo LF 2011 In vitro interaction between the ammonium transport protein AmtB and partially uridylylated forms of the PII protein GlnZ. Biochim Biophys Acta 1814:1203–1209. doi:10.1016/j.bbapap.2011.05.012. 21645649

[21] Araújo LM, Monteiro RA, Souza EM, Steffens MBR, Rigo LU, Pedrosa FO, Chubatsu LS 2004 GlnB is specifically required for *Azospirillum brasilense* NifA activity in *Escherichia coli*. Res Microbiol 155:491–495. doi:10.1016/j.resmic.2004.03.002. 15249067

[22] Huergo LF, Merrick M, Monteiro RA, Chubatsu LS, Steffens MBR, Pedrosa FO, Souza EM 2009 In vitro interactions between the PII proteins and the nitrogenase regulatory enzymes dinitrogenase reductase ADP-ribosyltransferase (DraT) and dinitrogenase reductase-activating glycohydrolase (DraG) in *Azospirillum brasilense*. J Biol Chem 284:6674–6682. doi:10.1074/jbc.M807378200. 19131333 PMC2652340

[23] Moure VR, Costa FF, Cruz LM, Pedrosa FO, Souza EM, Li XD, Winkler F, Huergo LF 2014 Regulation of nitrogenase by reversible mono-ADP-ribosylation. Curr Top Microbiol Immunol 384:89–106. doi:10.1007/82_2014_380. 24934999

[24] Huergo LF, Souza EM, Steffens MBR, Yates MG, Pedrosa FO, Chubatsu LS 2003 Regulation of glnB gene promoter expression in *Azospirillum brasilense* by the NtrC protein. FEMS Microbiol Lett 223:33–40. doi:10.1016/S0378-1097(03)00346-X. 12798997

[25] Scholl J, Dengler L, Bader L, Forchhammer K 2020 Phosphoenolpyruvate carboxylase from the cyanobacterium *Synechocystis* sp. PCC 6803 is under global metabolic control by P II signaling. Mol Microbiol 114:292–307. doi:10.1111/mmi.14512. 32274833

[26] Santos ARS, Gerhardt ECM, Parize E, Pedrosa FO, Steffens MBR, Chubatsu LS, Souza EM, Passaglia LMP, Sant'Anna FH, de Souza GA, Huergo LF, Forchhammer K 2020 NAD^+^ biosynthesis in bacteria is controlled by global carbon/nitrogen levels via PII signaling. J Biol Chem 295:6165–6176. doi:10.1074/jbc.RA120.012793. 32179648 PMC7196632

[27] Rodionova IA, Goodacre N, Babu M, Emili A, Uetz P, Saier MH 2017 The nitrogen regulatory PII protein (GlnB) and N-acetylglucosamine 6-phosphate epimerase (NanE) allosterically activate glucosamine 6-phosphate deaminase (NagB) in *Escherichia coli*. J Bacteriol 200:e00691-17. doi:10.1128/JB.00691-17. PMC5809692 29229699

[28] Rodrigues TE, Gerhardt ECM, Oliveira MA, Chubatsu LS, Pedrosa FO, Souza EM, Souza GA, Müller-Santos M, Huergo LF 2014 Search for novel targets of the P_II_ signal transduction protein in Bacteria identifies the BCCP component of acetyl-CoA carboxylase as a PII binding partner. Mol Microbiol 91:751–761. doi:10.1111/mmi.12493. 24329683

[29] Chellamuthu VR, Alva V, Forchhammer K 2013 From cyanobacteria to plants: conservation of PII functions during plastid evolution. Planta 237:451–462. doi:10.1007/s00425-012-1801-0. 23192387

[30] Bourrellier ABF, Valot B, Guillot A, Ambard-Bretteville F, Vidal J, Hodges M 2010 Chloroplast acetyl-CoA carboxylase activity is 2-oxoglutarate-regulated by interaction of PII with the biotin carboxyl carrier subunit. Proc Natl Acad Sci U S A 107:502–507. doi:10.1073/pnas.0910097107. 20018655 PMC2806706

[31] Gerhardt ECM, Rodrigues TE, Müller-Santos M, Pedrosa FO, Souza EM, Forchhammer K, Huergo LF 2015 The bacterial signal transduction protein GlnB regulates the committed step in fatty acid biosynthesis by acting as a dissociable regulatory subunit of acetyl-CoA carboxylase. Mol Microbiol 95:1025–1035. doi:10.1111/mmi.12912. 25557370

[32] Hauf W, Schmid K, Gerhardt ECM, Huergo LF, Forchhammer K 2016 Interaction of the nitrogen regulatory protein GlnB (PII) with biotin carboxyl carrier protein (BCCP) controls acetyl-CoA levels in the cyanobacterium *Synechocystis* sp. PCC 6803. Front Microbiol 7:1700. doi:10.3389/fmicb.2016.01700. 27833596 PMC5080355

[33] Tong L 2013 Structure and function of biotin-dependent carboxylases. Cell Mol Life Sci 70:863–891. doi:10.1007/s00018-012-1096-0. 22869039 PMC3508090

[34] Xu M, Tang M, Chen J, Yang T, Zhang X, Shao M, Xu Z, Rao Z 2020 PII signal transduction protein GlnK alleviates feedback inhibition of N-acetyl-l-glutamate kinase by l-arginine in *Corynebacterium glutamicum*. Appl Environ Microbiol 86:e00039-20. doi:10.1128/AEM.00039-20. 32060028 PMC7117919

[35] Ehlers C, Weidenbach K, Veit K, Forchhammer K, Schmitz RA 2005 Unique mechanistic features of post-translational regulation of glutamine synthetase activity in *Methanosarcina mazei* strain Gö1 in response to nitrogen availability. Mol Microbiol 55:1841–1854. doi:10.1111/j.1365-2958.2005.04511.x. 15752204

[36] Pedro-Roig L, Camacho M, Bonete MJ 2013 Regulation of ammonium assimilation in *Haloferax mediterranei*: interaction between glutamine synthetase and two GlnK proteins. Biochim Biophys Acta 1834:16–23. doi:10.1016/j.bbapap.2012.10.006. 23069245

[37] Potrykus K, Cashel M 2008 (p)ppGpp: still magical? Annu Rev Microbiol 62:35–51. doi:10.1146/annurev.micro.62.081307.162903. 18454629

[38] Brown DR, Barton G, Pan Z, Buck M, Wigneshweraraj S 2014 Nitrogen stress response and stringent response are coupled in *Escherichia coli*. Nat Commun 5:4115. doi:10.1038/ncomms5115. 24947454 PMC4066584

[39] Mackie GA 2013 RNase E: at the interface of bacterial RNA processing and decay. Nat Rev Microbiol 11:45–57. doi:10.1038/nrmicro2930. 23241849

[40] Bologna FP, Andreo CS, Drincovich MF 2007 *Escherichia coli* malic enzymes: two isoforms with substantial differences in kinetic properties, metabolic regulation, and structure. J Bacteriol 189:5937–5946. doi:10.1128/JB.00428-07. 17557829 PMC1952036

[41] Wang B, Wang P, Zheng E, Chen X, Zhao H, Song P, Su R, Li X, Zhu G 2011 Biochemical properties and physiological roles of NADP-dependent malic enzyme in *Escherichia coli*. J Microbiol 49:797–802. doi:10.1007/s12275-011-0487-5. 22068497

[42] Huergo LF, Araújo GA, Santos AS, Gerhardt ECM, Pedrosa FO, Souza EM, Forchhammer K 2020 The NADP-dependent malic enzyme MaeB is a central metabolic hub controlled by the acetyl-CoA to CoASH ratio. Biochim Biophys Acta Proteins Proteom 1868:140462. doi:10.1016/j.bbapap.2020.140462. 32485238

[43] Lee C-R, Cho S-H, Yoon M-J, Peterkofsky A, Seok Y-J 2007 *Escherichia coli* enzyme IIANtr regulates the K+ transporter TrkA. Proc Natl Acad Sci U S A 104:4124–4129. doi:10.1073/pnas.0609897104. 17289841 PMC1794712

[44] Lee C-R, Park Y-H, Kim M, Kim Y-R, Park S, Peterkofsky A, Seok Y-J 2013 Reciprocal regulation of the autophosphorylation of enzyme INtr by glutamine and α-ketoglutarate in *Escherichia coli*. Mol Microbiol 88:473–485. doi:10.1111/mmi.12196. 23517463 PMC3633653

[45] Coutts G, Thomas G, Blakey D, Merrick M 2002 Membrane sequestration of the signal transduction protein GlnK by the ammonium transporter AmtB. EMBO J 21:536–545. doi:10.1093/emboj/21.4.536. 11847102 PMC125854

[46] Yan D, Ikeda TP, Shauger AE, Kustu S 1996 Glutamate is required to maintain the steady-state potassium pool in *Salmonella typhimurium*. Proc Natl Acad Sci U S A 93:6527–6531. doi:10.1073/pnas.93.13.6527. 8692849 PMC39057

[47] Tautenhahn R, Patti GJ, Rinehart D, Siuzdak G 2012 XCMS online: a web-based platform to process untargeted metabolomic data. Anal Chem 84:5035–5039. doi:10.1021/ac300698c. 22533540 PMC3703953

[48] Lu W, Clasquin MF, Melamud E, Amador-Noguez D, Caudy AA, Rabinowitz JD 2010 Metabolomic analysis via reversed-phase ion-pairing liquid chromatography coupled to a stand alone orbitrap mass spectrometer. Anal Chem 82:3212–3221. doi:10.1021/ac902837x. 20349993 PMC2863137

[49] Jenal U, Reinders A, Lori C 2017 Cyclic di-GMP: second messenger extraordinaire. Nat Rev Microbiol 15:271–284. doi:10.1038/nrmicro.2016.190. 28163311

[50] O’Neal L, Ryu M-H, Gomelsky M, Alexandre G 2017 Optogenetic manipulation of cyclic di-GMP (c-di-GMP) levels reveals the role of c-di-GMP in regulating aerotaxis receptor activity in *Azospirillum brasilense*. J Bacteriol 199:e00020-17. doi:10.1128/JB.00020-17. 28264994 PMC5573079

[51] Hove-Jensen B, Andersen KR, Kilstrup M, Martinussen J, Switzer RL, Willemoës M 2017 Phosphoribosyl diphosphate (PRPP): biosynthesis, enzymology, utilization, and metabolic significance. Microbiol Mol Biol Rev 81:e00040-16. doi:10.1128/MMBR.00040-16. 28031352 PMC5312242

[52] Vázquez-Salazar A, Becerra A, Lazcano A 2018 Evolutionary convergence in the biosyntheses of the imidazole moieties of histidine and purines. PLoS One 13:e0196349. doi:10.1371/journal.pone.0196349. 29698445 PMC5919458

[53] Chen S, Liu L, Zhou X, Elmerich C, Li J-L 2005 Functional analysis of the GAF domain of NifA in *Azospirillum brasilense*: effects of Tyr→Phe mutations on NifA and its interaction with GlnB. Mol Genet Genomics 273:415–422. doi:10.1007/s00438-005-1146-5. 15887032

[54] Watzer B, Spät P, Neumann N, Koch M, Sobotka R, MacEk B, Hennrich O, Forchhammer K 2019 The signal transduction protein PII controls ammonium, nitrate and urea uptake in cyanobacteria. Front Microbiol 10:1428. doi:10.3389/fmicb.2019.01428. 31293555 PMC6603209

[55] Rajendran C, Gerhardt ECM, Bjelic S, Gasperina A, Scarduelli M, Pedrosa FO, Chubatsu LS, Merrick M, Souza EM, Winkler FK, Huergo LF, Li XD 2011 Crystal structure of the GlnZ-DraG complex reveals a different form of P II-target interaction. Proc Natl Acad Sci U S A 108:18972–18976. doi:10.1073/pnas.1108038108. 22074780 PMC3223478

[56] Huergo LF, Merrick M, Pedrosa FO, Chubatsu LS, Araujo LM, Souza EM 2007 Ternary complex formation between AmtB, GlnZ and the nitrogenase regulatory enzyme DraG reveals a novel facet of nitrogen regulation in bacteria. Mol Microbiol 66:1523–1535. doi:10.1111/j.1365-2958.2007.06016.x. 18028310

[57] O’Neal L, Mukherjee T, Alexandre G 2018 Analyzing chemotaxis and related behaviors of *Azospirillum brasilense*. Curr Protoc Microbiol 48:3E.3.1–3E.3.11. doi:10.1002/cpmc.49. 29512118

[58] El-Gebali S, Mistry J, Bateman A, Eddy SR, Luciani A, Potter SC, Qureshi M, Richardson LJ, Salazar GA, Smart A, Sonnhammer ELL, Hirsh L, Paladin L, Piovesan D, Tosatto SCE, Finn RD 2019 The Pfam protein families database in 2019. Nucleic Acids Res 47:D427–D432. doi:10.1093/nar/gky995. 30357350 PMC6324024

[59] Yuan X, Zeng Q, Xu J, Severin GB, Zhou X, Waters CM, Sundin GW, Ibekwe AM, Liu F, Yang C-H 2020 Tricarboxylic acid (TCA) cycle enzymes and intermediates modulate intracellular cyclic di-GMP levels and the production of plant cell wall–degrading enzymes in soft rot pathogen *Dickeya dadantii*. Mol Plant Microbe Interact 33:296–307. doi:10.1094/MPMI-07-19-0203-R. 31851880 PMC9354473

[60] Araujo MS, Baura VA, Souza EM, Benelli EM, Rigo LU, Steffens MBR, Pedrosa FO, Chubatsu LS 2004 In vitro uridylylation of the *Azospirillum brasilense* N-signal transducing GlnZ protein. Protein Expr Purif 33:19–24. doi:10.1016/j.pep.2003.08.024. 14680957

[61] Kukolj C, Pedrosa FO, De Souza GA, Sumner LW, Lei Z, Sumner B, Do Amaral FP, Juexin W, Trupti J, Huergo LF, Monteiro RA, Valdameri G, Stacey G, De Souza EM 2020 Proteomic and metabolomic analysis of *Azospirillum brasilense* ntrC mutant under high and low nitrogen conditions. J Proteome Res 19:92–105. doi:10.1021/acs.jproteome.9b00397. 31599156

[62] Santos ARS, Gerhardt ECM, Moure VR, Pedrosa FO, Souza EM, Diamanti R, Högbom M, Huergo LF 2018 Kinetics and structural features of dimeric glutamine-dependent bacterial NAD synthetases suggest evolutionary adaptation to available metabolites. J Biol Chem 293:7397–7407. doi:10.1074/jbc.RA118.002241. 29581233 PMC5950007

[63] Massie JP, Reynolds EL, Koestler BJ, Cong JP, Agostoni M, Waters CM 2012 Quantification of high-specificity cyclic diguanylate signaling. Proc Natl Acad Sci U S A 109:12746–12751. doi:10.1073/pnas.1115663109. 22802636 PMC3411991

[64] Neyra CA, Van Berkum P 1977 Nitrate reduction and nitrogenase activity in *Spirillum lipoferum*. Can J Microbiol 23:306–310. doi:10.1139/m77-045. 856423

[65] Martens L, Hermjakob H, Jones P, Adamsk M, Taylor C, States D, Gevaert K, Vandekerckhove J, Apweiler R 2005 PRIDE: the proteomics identifications database. Proteomics 5:3537–3545. doi:10.1002/pmic.200401303. 16041671

[66] Machado HB, Yates MG, Funayama S, Rigo LU, Steffens MBR, Souza EM, Pedrosa FO 1995 The *ntrBC* genes of *Azospirillum brasilense* are part of a *nifR3*-like–*ntrB*–*ntrC* operon and are negatively regulated. Can J Microbiol 41:674–684. doi:10.1139/m95-093. 7553451

[67] Vanstockem M, Michiels K, Vanderleyden J, Van Gool AP 1987 Transposon mutagenesis of *Azospirillum brasilense* and *Azospirillum lipoferum*: physical analysis of Tn*5* and Tn*5*-Mob insertion mutants. Appl Environ Microbiol 53:410–415. doi:10.1128/aem.53.2.410-415.1987. 16347289 PMC203674

