# Supplementary figures and images for: The Protein-Protein Interaction Network Reveals a Novel Role of the Signal Transduction Protein PII in the Control of c-di-GMP Homeostasis in Azospirillum brasilense
Source: mSystems. 2020 Nov 3;5(6):e00817-20. doi: 10.1128/mSystems.00817-20 (PMC7646526; doi:10.1128/mSystems.00817-20)

Figure S1

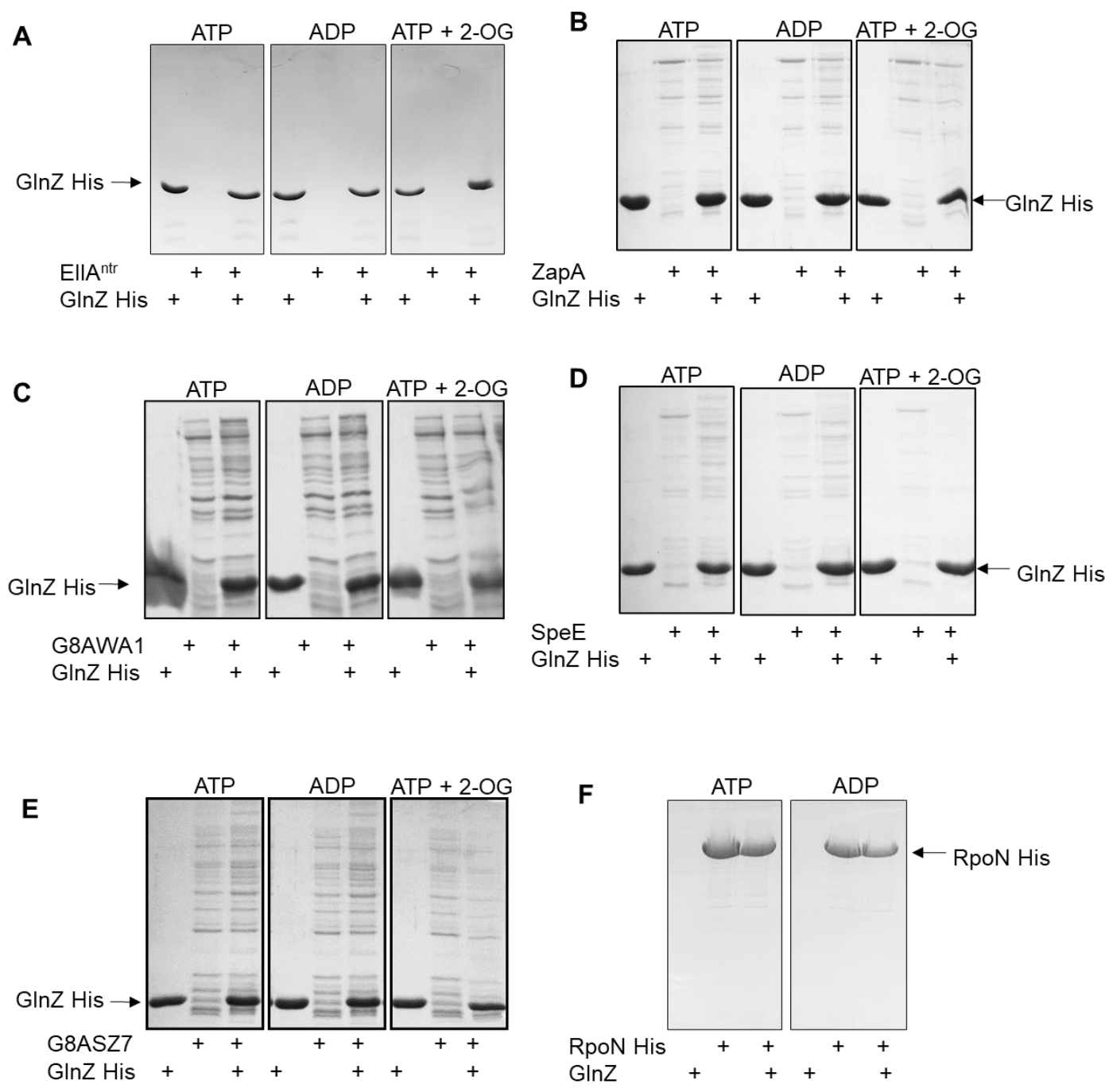

Supplement: FIG S1 [file mSystems.00817-20-sf001.pdf]

Figure S2

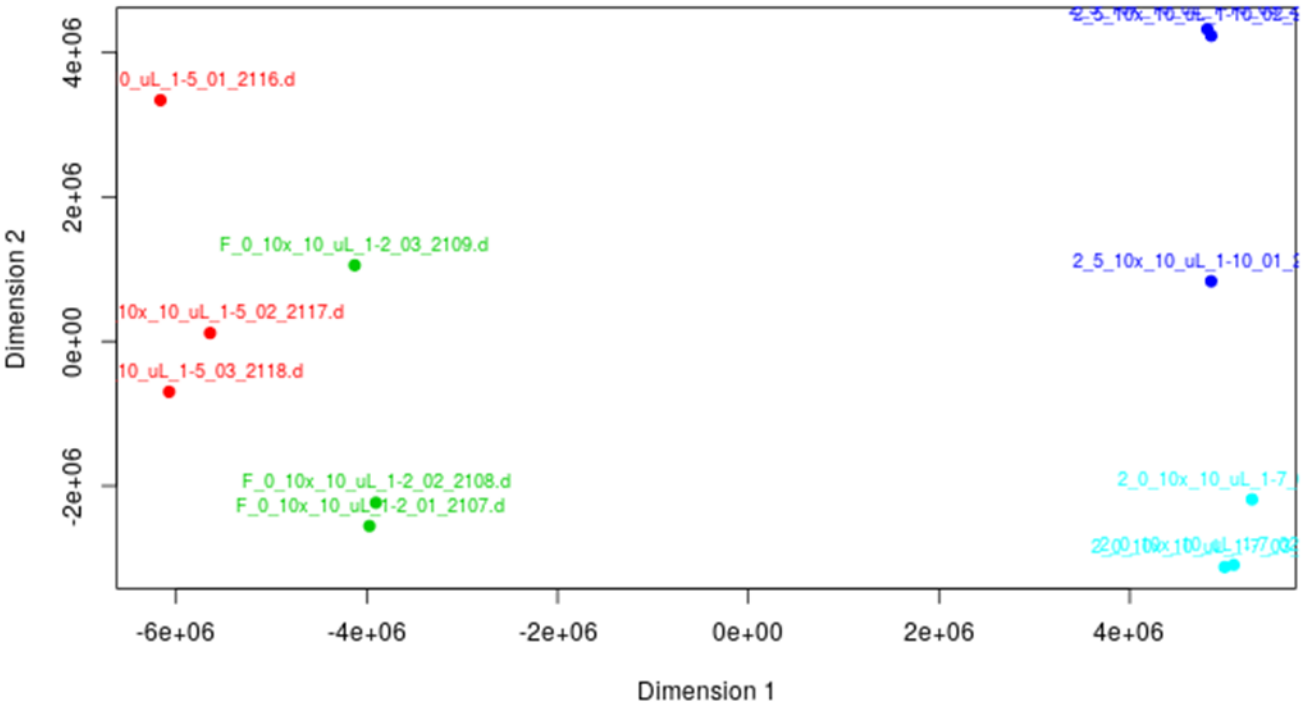

Supplement: FIG S2 [file mSystems.00817-20-sf002.pdf]

Figure S3

A

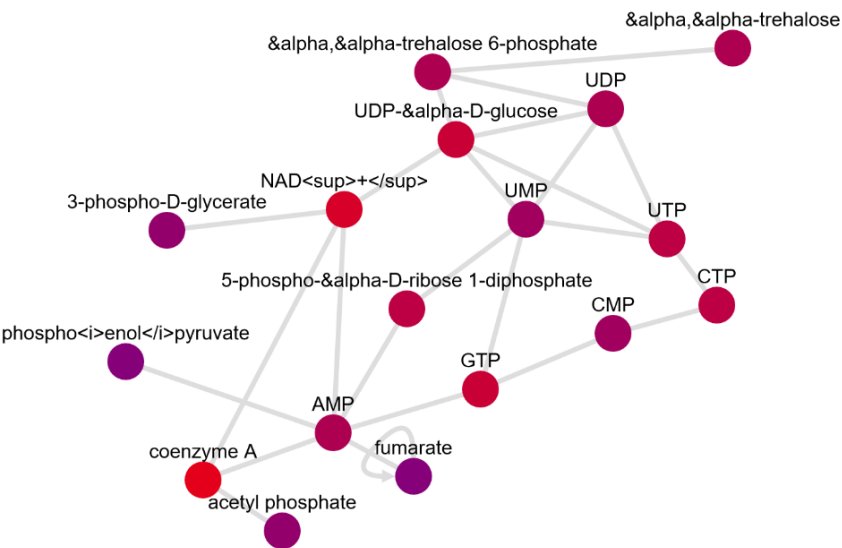

B

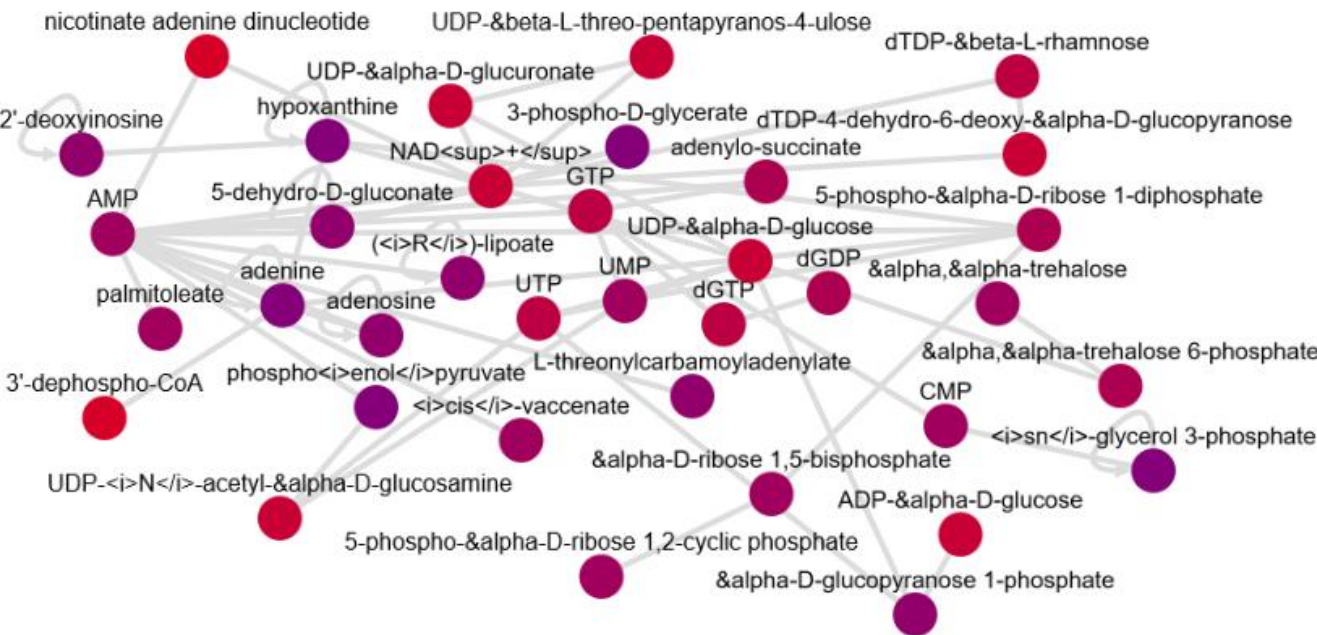

Supplement: FIG S3 [file mSystems.00817-20-sf003.pdf]

Figure S4

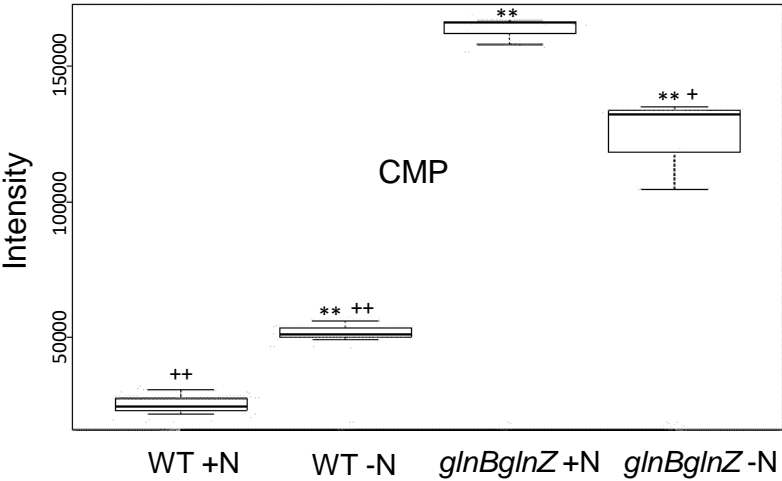

Supplement: FIG S4 [file mSystems.00817-20-sf004.pdf]
