# Supplementary material for: The Protein-Protein Interaction Network Reveals a Novel Role of the Signal Transduction Protein PII in the Control of c-di-GMP Homeostasis in Azospirillum brasilense
Source: mSystems. 2020 Nov 3;5(6):e00817-20. doi: 10.1128/mSystems.00817-20 (PMC7646526; doi:10.1128/mSystems.00817-20)
Supplement: TEXT S1 [file mSystems.00817-20-s0001.docx]

**Supplementary text S1**

**General Molecular Biology and protein methods**

Isolation of plasmid DNA, gel electrophoresis, bacterial transformation and cloning were performed as described (Sambrook et al., 1989). Enzymes were obtained from commercial sources and used according to the manufacturers’ instructions. DNA sequencing was performed using dye-labeled terminators (ET terminator – GE healthcare) in an automated DNA sequencer ABI 3500 from Applied Biosystems. Electrophoresis of proteins was carried out by SDS-PAGE and gels were Coomassie stained. Protein concentrations were determined by the Bradford assay using bovine serum albumin as standard.

**Construction of vector to express N-terminal 3xFLAG GlnZ**

The *glnZ* coding sequence (GenBank X92496.1) containing a 3x N-terminal FLAG tag and flanked by *Nde*I and *Eco*RI was codon optimized, synthesized by Eurofins (Germany) and cloned into the pET29a vector (Novagen) by using the Gibson method (1). The construction was confirmed by DNA sequencing.

**Construction of vectors to express the putative PII-target proteins**

The coding sequence for the *A. brasilense* genes AZOBR_140132 (NCBI_ID WP_014240625; Uniprot G8AME0), AZOBR_p1130052 (NCBI_ID WP_014198291, Uniprot G8ATR8), *puuA* (WP_014240470.1, Uniprot G8ALX8), *relA* (WP_014198736.1, Uniprot G8APW7) and *zapA* (WP_014240504.1, Uniprot G8AM13) were codon optimized, synthesized and cloned into the pET29a vector to express untagged proteins by General Biosystems (USA) (Table S3). The remaining genes were PCR amplified using the primers listed in Table S3, proofreading *pfu* DNA polymerase (Agilent) and genomic DNA from *A. brasilense* FP2 as a template. PCR amplicons were cloned into the pET28a, pET29a (Agilent) and/or pTEV5 (2) using the restriction sites *Nde*I – *Bam*HI or *Nde*I – *EcoR*I as indicated in Table S3.

**In gel protein digestion and MALDI-TOF mass spectrometry analysis**

The eluted fraction from the GlnZ ligand fishing assays performed in the presence of ATP were analyzed by SDS-PAGE. Protein bands that were visually considered enriched in the fractions eluted from the GlnZ affinity columns in comparison to the background control were excised from Coomassie stained SDS-PAGE gels and subjected to in-gel digestion with sequencing-grade trypsin as described (3). For MALDI-TOF analysis the hydrolyzed sample was mixed with a saturated matrix solution of α-cyano-4-hydroxycinnamic acid (dissolved in acetonitrile 50% v/v, TFA 0.1% v/v), spotted onto the MALDI target and allowed to dry. Mass spectra were acquired using a MALDI-TOF/TOF Autoflex II spectrometer (Bruker Daltonics). MS analyses were performed in a positive ion reflection mode using accelerating voltage of 20kV. Peak lists were created using the FlexAnalysis 3.0 software (Bruker Daltonics). Database search was performed using Mascot 2.2 server (Matrix Science). Mass lists were searched against a database of *A. brasilense* downloaded from Uniprot. Error tolerance was 100 ppm for peptide mass fingerprint search.

**Label free quantitative LC-MS/MS analysis**

Proteins eluted from His-tagged GlnZ Ni^2+^ affinity columns or FLAG-tagged GlnZ affinity columns were analyzed by label free LC-MS/MS as described previously (4). Briefly, aliquots of 15 µg of the GlnZ affinity column and from the respective control column were suspended in 50 μl of ammonium bicarbonate 100 mM pH 8.0. Proteins were reduced with DTT 1 mM for 45 min. Modified trypsin (Promega) was added to a 1:50 ratio and incubated overnight at 37 °C. Protein digestion was quenched with TFA 3% (v/v), peptides were extracted with C18 STAGE-TIPs and subjected to technical triplicate LC/MS/MS runs. Samples were analyzed in a QExactive Orbitrap (Thermo Scientific) and the data was processed using MaxQuant v1.5.2.8 (5). Proteins were identified using an *A. brasilense* protein database downloaded from Uniprot. Statistical analysis was performed using MaxQuant – Perseus package version 1.5.0.30 and statistically significant differences were assigned using a One-way ANOVA test with p-value threshold of 0.05 and Benjamin Hochberg-based FDR correction. Proteins exhibiting more than 3x enrichment and p<0.05 in the samples eluted from the GlnZ affinity column in comparison to the respective control were considered as potential GlnZ-binding partners.

**Recombinant protein expression and purification**

The genes encoding the protein of interest were cloned into T7 promoter expression vectors as described in Table S3. Recombinant protein expression was performed using *E. coli* BL21 (λDE3) after induction with IPTG. The *A. brasilense* N-terminal 6xHis-tag GlnZ was expressed and purified as described previously (6). All other His tag protein were purified using a similar protocol. The untagged proteins used in this study were purified using a combination of different chromatography resins, the detailed protocols for the purification of each protein are available upon request. In some cases, for protein complex pull-down analysis, the total soluble protein extract of *E. coli* BL21 (λDE3) overexpressing the recombinant protein was used when indicated in each experiment.

**Protein complex pull-down assays using nickel magnetic beads**

*In vitro* protein complex formation was assessed using MagneHis beads (Promega) as described (7). All reactions were conducted in buffer containing 50 mM Tris-HCl pH 8, 0.1 M NaCl, 10% glycerol and 20 mM imidazole in the presence of PII effectors as indicated in each experiment. Five microliters of beads were equilibrated by three washes with 300 μl of buffer. Binding reactions were performed in 500 μl of buffer by adding 20 µg of His-GlnZ as bait and 40 µg of purified GlnZ-target protein. When indicated, 100 µl of the total soluble protein extract of *E. coli* BL21 (λDE3) overexpressing the recombinant protein was used as a source of the GlnZ-target protein instead. Purified GlnZ and target proteins were incubated with the beads for 15 min, beads were washed 3 times with 300 μl of buffer. Elution was performed with 20 μl of SDS-PAGE sample buffer at 95°C for 5 min. Proteins were analyzed in 15% SDS-PAGE stained with Coomassie blue.

**Untargeted metabolomic analysis of *A. brasilense***

The *A. brasilense* wild-type FP2 or 2812 (PII double mutant *glnBglnZ*) strains were cultured in triplicate vials in NFbHP medium containing 20 mM NH_4_Cl at 30ºC, 120 rpm until an O.D_600_ of 1. Cells were collected by centrifugation (2,000 xg, 10 minutes 4ºC), resuspended and maintained for 2 hours at 30ºC, 120 rpm in NFbHP without fixed nitrogen aerobically in order to obtain nitrogen starved cells (-N) and induce PII protein expression. Aliquots of the cell cultures (15 ml) were collected before (-N) and 5 min after the addition of NH_4_Cl 1mM (+N). The tubes containing the cells were immediately chilled by submersion in liquid nitrogen and centrifuged at 20,000 xg for 30 seconds at 4ºC. Metabolites were extracted using 300 µl of extraction solvent (40:40:20 acetonitrile:methanol:water; 0.1M formic acid) at -20ºC. The mixture was kept on ice for 10 min and then centrifuged 20,000 xg, 5 min at 4ºC). Two hundred microliters of supernatant were transferred to a new tube. This extraction procedure was repeated, the two supernatants were combined and neutralized adding 22 µl of ammonium hydroxide (28-30%, Sigma Aldrich) followed by centrifugation (20,000 xg, 20 min, 4ºC). The final supernatant was transferred to vials and 10 µl were subjected to LC-MS analysis.

Metabolites were separated using a UFLC Prominence (Shimadzu) in a C18 column 2.6 µm 50 × 2.1 mm (Phenomenex) kept at 40 °C. The mobile phases were composed of 15 mM acetic acid and 10 mM tributylamine (solvent A) or methanol (solvent B). Samples were placed into vials and kept in an auto-sampler at 4 °C; 10 µl of each sample was injected at 0.2 ml.min^−1^. Metabolites were eluted from the column using a linear gradient (0–100 % of solvent B) in 30 min. The detection of the metabolites was performed by coupling the LC with a MicroTOF-QII (Bruker Daltonics), equipped with an electrospray ionization source operating at negative mode, scanning 50 to 1000 m/z each second. The LC-MS data generated from independent triplicate samples were analyzed using XCMS online (8). Feature detection set to: 5 ppm; minimum and maximum peak width 10 and 30, respectively; signal/noise threshold 20, m/z diff 0.03. Feature annotation was performed using 10 ppm, absolute error of 0.02. The identity of some metabolites was further investigated by comparing the elution profile with those from authenticated standards described in the literature.

**Quantification of c-di-GMP levels *in vivo***

The *A. brasilense* strains wild-type Sp7 and 7611 (*glnZ* minus) were cultured in minimal medium containing 20 mM NH_4_Cl and 10 mM L-malate as carbon source to O.D_600_ of 0.5. Cells were washed and resuspended in media containing 10 mM L-malate without nitrogen to induce nitrogen fixation and GlnZ protein expression. The cultures were split into 2x 10 ml samples. The c-di-GMP was extracted from one 10 ml sample to establish basal c-di-GMP levels (nitrogen fixation condition). The other 10 ml sample was subjected to an ammonium shock (20mM NH_4_Cl) for 30 min before c-di-GMP extraction. In third condition, named carbon starvation, cells were incubated for 30 min in media containing 1 mM L-malate and 20 mM NH_4_Cl before c-di-GMP extraction. The 10 ml cell aliquots were collected by centrifugation, pellets were weighed and treated using 100 µl of solvent (40% methanol, 40% acetonitrile, and 20% 0.1 N formic acid) per 0.048g of pellet. Extraction slurries were incubated at -20ºC for 30 minutes. Insoluble material was removed by centrifugation at 20,000 xg, 3 min at 4ºC. Supernatant was removed and neutralized with 4 µl of 15% ammonium bicarbonate per 100 µl of supernatant. The extraction was repeated on the insoluble material and the neutralized supernatants were combined and dried for 2 hours using a speed vac. Samples were shipped overnight to MSU Mass spectrometry and metabolomics facility were c-di-GMP was quantified by LC/MS/MS, as described (9).

**Cell swimming aerotaxis assay**

The *A. brasilense* strains were grown and prepared as previously described (10). The cells were gently washed three times with chemotaxis buffer (K_2_HPO_4_ 1.7 g.l^-1^, KH_2_PO_4_ 1.36 g.l^-1^) by low-speed centrifugation and resuspended in 100 μl MMAB containing malate (10 mM). All cells remained motile under these conditions. An optically flat capillary tube (Vitro Dynamics, Inc., Rockaway, NJ) was placed in the bacterial culture for 10 s allowing the cell suspension to fill the tube by capillarity. The tubes containing the cell suspensions were then placed in a perfusing chamber and equilibrated with humidified N_2_ for 2 min. After equilibration, nitrogen gas was turned off and air was allowed to flow into the chamber thereby forming an oxygen gradient. Aerotaxis was visualized using the 4x objective of a phase contrast Nikon E200 microscope. Images were acquired using a C-mounted Nikon Coolpix digital camera. The distance of the biomass band from the end of the capillary tube was measured using ImageJ software. All measurements were performed at 120 s after N_2_ shut off (10).

**Flocculation assay**

The *A. brasilense* strains were grown in 5 ml of MMAB supplemented with nitrogen (20 mM) and malate (10 mM) to O.D_600_ = 0.8. Cells were centrifuged and washed with chemotaxis buffer (K_2_HPO_4_ 1.7 g.l^-1^, KH_2_PO_4_ 1.36 g.l^-1^). Culture were re-suspended to a final O.D_600_= 0.16 in 1 ml of flocculation medium (11) containing 0.5 mM NH_4_Cl and 8 mM malate. Cultures were placed in 24-well plates and grown at room temperature for 24 h, with shaking at 60 rpm. Supernatants of cell suspensions (therefore excluding flocculated cells that fell at the bottom of the wells) were transferred to cuvettes to measure O.D_600_.

**References**

1. Gibson DG, Young L, Chuang RY, Venter JC, Hutchison CA, Smith HO. 2009. Enzymatic assembly of DNA molecules up to several hundred kilobases. Nat Methods.

2. Rocco CJ, Dennison KL, Klenchin VA, Rayment I, Escalante-Semerena JC. 2008. Construction and use of new cloning vectors for the rapid isolation of recombinant proteins from *Escherichia coli*. Plasmid.

3. Huergo LF, Noindorf L, Gimenes C, Lemgruber RSP, Cordellini DF, Falarz LJ, Cruz LM, Monteiro RA, Pedrosa FO, Chubatsu LS, Souza EM, Steffens MBR. 2010. Proteomic analysis of *Herbaspirillum seropedicae* reveals ammonium-induced AmtB-dependent membrane sequestration of PII proteins. FEMS Microbiol Lett 308:40–47.

4. Kukolj C, Pedrosa FO, De Souza GA, Sumner LW, Lei Z, Sumner B, Do Amaral FP, Juexin W, Trupti J, Huergo LF, Monteiro RA, Valdameri G, Stacey G, De Souza EM. 2020. Proteomic and Metabolomic Analysis of *Azospirillum brasilense* ntrC Mutant under High and Low Nitrogen Conditions. J Proteome Res.

5. Cox J, Mann M. 2008. MaxQuant enables high peptide identification rates, individualized p.p.b.-range mass accuracies and proteome-wide protein quantification. Nat Biotechnol.

6. Huergo LF, Merrick M, Pedrosa FO, Chubatsu LS, Araujo LM, Souza EM. 2007. Ternary complex formation between AmtB, GlnZ and the nitrogenase regulatory enzyme DraG reveals a novel facet of nitrogen regulation in bacteria. Mol Microbiol 66.

7. Huergo LF, Merrick M, Monteiro RA, Chubatsu LS, Steffens MBR, Pedrosa FO, Souza EM. 2009. In vitro interactions between the PII proteins and the nitrogenase regulatory enzymes dinitrogenase reductase ADP-ribosyltransferase (DraT) and dinitrogenase reductase-activating glycohydrolase (DraG) in *Azospirillum brasilense*. J Biol Chem 284.

8. Tautenhahn R, Patti GJ, Rinehart D, Siuzdak G. 2012. XCMS online: A web-based platform to process untargeted metabolomic data. Anal Chem.

9. Massie JP, Reynolds EL, Koestler BJ, Cong JP, Agostoni M, Waters CM. 2012. Quantification of high-specificity cyclic diguanylate signaling. Proc Natl Acad Sci U S A.

10. O’Neal L, Mukherjee T, Alexandre G. 2018. Analyzing Chemotaxis and Related Behaviors of *Azospirillum brasilense*. Curr Protoc Microbiol.

11. Neyra CA, Van Berkum P. 1977. Nitrate reduction and nitrogenase activity in *Spirillum lipoferum.* Can J Microbiol.
