# Supplementary material for: The Protein-Protein Interaction Network Reveals a Novel Role of the Signal Transduction Protein PII in the Control of c-di-GMP Homeostasis in Azospirillum brasilense
Source: mSystems. 2020 Nov 3;5(6):e00817-20. doi: 10.1128/mSystems.00817-20 (PMC7646526; doi:10.1128/mSystems.00817-20)
Supplement: TABLE S3 [file mSystems.00817-20-st003.docx]

| **Table S3** | | | | | |
| --- | --- | --- | --- | --- | --- |
| **Synthetic genes (all genes were codon optimized for *E. coli* expression and cloned into pET29a *Nde*I – *Bam*HI to express untagged proteins** | | | | | |
| NCBI_ID | Gene | Uniprot-ID | Protein | Source | Plasmid |
| WP_014240625 | AZOBR_140132 | G8AME0 | putative diguanylate cyclase, GGDEF domain | General Biosystems | pET29a |
| WP_014198291 | AZOBR_p1130052 | G8ATR8 | putative Diguanylate cyclase/ phosphodiesterase with PAS/PAC domain | General Biosystems | pET29a |
| WP_014240470.1 | *puuA* | G8ALX8 | Gamma-Glu-putrescine synthase | General Biosystems | pET29a |
| WP_014198736.1 | *relA* | G8APW7 | ppGpp sinthetase | General Biosystems | pET29a |
| WP_014240504.1 | *zapA* | G8AM13 | Cell division protein ZapA | General Biosystems | pET29a |
| WP_014199773 | *ptsI* | G8AZB2 | Phosphoenolpyruvate-protein phosphotransferase | General Biosystems | pET29a |
| HE577328 | AZOBR*_*p1170085 | G8APW6 | Uncharacterized protein ( DNA-directed RNA polymerase subunit omega) (RpoZ) | General Biosystems | pET29a |
| **Primers (primers were purchased from IDT)** | | | | | |
| NCBI_ID | Gene | Uniprot-ID | Protein | Primers (5'-3') |  |
| WP_014198053 | *rpoN* (AZOBR_p1110045) | G8AT20 | RNA polymerase sigma-54 factor (RpoN) | F CCCTGCATATGGCGCTCAGCCAACGC |  |
|  |  |  |  | R CGACCGGATCCCGGCCCGGTTACATGCGT |  |
| WP_014239675 | *pyr*E2 (AZOBR_70015) | G8AJG7 | orotate phosphoribosyltransferase (PyrE2) | F GTTAGCATATGACCACCGCCGCTTTGC |  |
|  |  |  |  | R CTCCGGGATCCTCAGTCGAAGCTGGCCTTGC |  |
| WP_014239021 | *Rph* (AZOBR_10486) | G8AGG4 | Ribonuclease PH (Rph) | F CATGCCATATGCGTCCCTCCGGC |  |
|  |  |  |  | R CGCCTGGATCCTTACTTCACCGCGATCGC |  |
| WP_014238784 | *mae*B1 (AZOBR_10249) | G8AFB3 | NADP-dependent malic enzyme (MaeB1) | F GAACCATATGACCGAACCCGATACAAAGC |  |
|  |  |  |  | R CCGCCGGATCCTCACCAGGGCAGCGAGT |  |
| WP_014239273 | *arg*B (AZOBR_40136) | G8AI64 | Acetylglutamate kinase (NAGK) | F TCCATCATATGCAGAACACGACCCGC |  |
|  |  |  |  | R CGCTTGGATCCTCACTCGCGGCCGATCA |  |
| WP_041812749 | *nadE1* | G8ASI0 | Glutamine-dependent NAD synthetase (NadE1) | F GGATCCATATGACGGTTGACGGCG |  |
|  |  |  |  | R GGGCGGATCCTTACTCCTTGGGCAC |  |
| WP_014199776 | AZOBR_p430053 | G8AZB5 | PTS system permease (IIAMan), nitrogen regulatory IIA protein (EIIA^Ntr^) | F GATGTCATATGATCGGTATGGTTCTGGTAACC |  |
|  |  |  |  | R CGTCTGGATCCTTCATCGGGTCACCCGTC |  |
| WP_014197050.1 | *trkA* (AZOBR_p110016) | G8AQ29 | Trk system potassium uptake protein (TrkA) | F CAAAGCATATGAAGGTCATCGTTTGCGGAG |  |
|  |  |  |  | R ACGGGAAGCTTTCAGAAGAATTCAAGCCGGACG |  |
| WP_014238917.1 | *rho* (AZOBR_10382) | G8AG60 | Transcription termination factor Rho (Rho)_ | F CCCCCCATATGCATCTCCAAGAGCTGAAG |  |
|  |  |  |  | R GCGGTAAGCTTCTATTGGTTCATGGACTCGAAGT |  |
| WP_035672384 | *glnE* | A7Y9V0 | Bifunctional glutamine synthetase adenylyltransferase/adenylyl-removing enzyme (ATase) | F CCGCGCATATGACCACCACGCTGCC |  |
|  |  |  |  | R GCGTCGGATCCTCATGGGAGTTTGGCTTCTTC |  |
| WP_014238768 | *coaBC* | G8AF97 | Coenzyme A biosynthesis bifunctional protein (CoaBC) | F CAAGTCATATGGCCGAAACATCCGTGC |  |
|  |  |  |  | R CGAGCGGATCCCGTTGCGCTCACGGAATG |  |
| WP_014242549.1﻿ | AZOBR_p280115 | G8AWC0 | Putative signal transduction histidine kinase | F AACGCCATATGGAACCGACCGAGTTTAAGC |  |
|  |  |  |  | R GAGAAGGATCCTCATCCGTTCCACTCCGC |  |
| WP_041811693.1 | AZOBR_100259 | G8AKW1 | Putative hydrolase of the metallo-beta-lactamase superfamily/Putative Ribonuclease J | F ACGGCCATATGACCCATCCCGACTCCG |  |
|  |  |  |  | R AAACTGGATCCCGCTTATACACGGACGAGG |  |
| WP_137139543.1 | *speE* | G8AGC5 | Polyamine aminopropyltransferase (SpeE) | F ACCGGCATATGAGCGACTCCGGCTC |  |
|  |  |  |  | R CGCCAGGATCCCTACTTCAGCGCGTCCAGC |  |
| CCD00558 | AZOBR*_*p1110022 | G8ASZ7 | 3-oxoacyl-(Acyl carrier protein) reductase | F GGCCCCATATGGATTTCCGCGACAAGAC |  |
|  |  |  |  | R GGGTGGGATCCAAGGGTCACCCCATCACCAA |  |
| CCD02210 | AZOBR_p280096 | G8AWA1 | Short-chain dehydrogenase/reductase | F CAGGACATATGACTTTGTTGGGCGAGGA |  |
|  |  |  |  | R CCCGCGGATCCTCACGCCATAAGCTGGCC |  |

| **Strains** | | |
| --- | --- | --- |
| **Strain/plasmid** | **Genotype/phenotype** | **Source/reference** |
| *A.  brasilense* |  |  |
| Sp7 | Wild-type | (1) |
| FP2 | Wild-type Sp7 isogenic | (1) |
| 2812 | *glnB::kan glnZ::Ω* Sp7 isogenic | (2) |
| 7611 | *glnZ::Ω* Sp7 isogenic | (2) |
| *E . coli* |  |  |
| DH10B | Sm^r^ ; F^’^ [*pro*AB^+^ *lacZ*M15] | Invitrogen |
| BL21 (λDE3) | Expresses T7 RNA Polymerase | Agillent |
| **Plasmids** | | |
| **Plasmid** | **Characteristics** | **Reference** |
| pET28a | Km^r^ Expression vector | Agilent |
| pET29a | Km^r^ Expression vector | Agilent |
| pTEV5 | Km^r^ Expression vector, His tag followed by TEV cleavage site | (3) |
| pMSA3 | Km^r^. Express *A. brasilense* GlnZ with a N-terminal 6x His tag in pET29a | (4) |
| pLHPZHISDK7 | Cm^r^. Express *A. brasilense* GlnZ with a N-terminal 6x His tag in pDK7 | (5) |
| pEGGlnZFlag | Km^r^ (pET29a). Express *A. brasilense* GlnZ subunit with 3x flag tag in N-terminal | This work |
| pLHpet29abDGC | Km^r^ (pET29a). Express *A. brasilense* G8AME0 (putative diguanylate cyclase, GGDEF domain) | This work |
| pLHpet29abPDE | Km^r^ (pET29a). Express *A. brasilense* G8ATR8 (putative Diguanylate cyclase/phosphodiesterase with PAS/PAC domain) | This work |
| pLHpet29abPuuA | Km^r^ (pET29a). Express *A. brasilense* G8ALX8 (Gamma-Glu-putrescine synthase) PuuA | This work |
| pLHpet29abRelA | Km^r^ (pET29a). Express *A. brasilense* G8APW7 (ppGpp synthetase) RelA | This work |
| pLHpet29abZapA | Km^r^ (pET29a). Express *A. brasilense* G8AM13 (Cell division protein ZapA) ZapA | This work |
| pAUpet28rpoN | Km^r^ (pET28a). Express *A. brasilense* G8AT20 (RNA polymerase sigma-54 factor) with a N-terminal 6x His tag RpoN | This work |
| pAGpet29pyrE | Km^r^ (pET29a). Express *A. brasilense* G8AJG7 (orotate phosphoribosyltransferase) PyrE2 | This work |
| pAGpet29rpH | Km^r^ (pET29a). Express *A. brasilense* G8AGG4 (Ribonuclease PH) Rph | This work |
| pGAMaeB1 | Km^r^ (pET29a). Express *A. brasilense* G8AFB3 (NADP-dependent malic enzyme) MaeB1 | (6) |
| pGApet29nagK | Km^r^ (pET29a). Express *A. brasilense* G8AI64 (N-Acetylglutamate kinase) NAGK | This work |
| pASnade1 | Amp^r^ (pTEV5). Express *A. brasilense* G8ASI0 (Glutamine-dependent NAD synthetase) NadE1 | (7) |
| pEGpt29IIAman | Km^r^ (pET29a). Express *A. brasilense* G8AZB5 (PTS system permease IIAMan) EIIA‑ | This work |
| pFG29-TrkA | Km^r^ (pET29a). Express *A. brasilense* G8AQ29 (Trk system potassium uptake protein) TrkA | This work |
| pFG29-Rho | Km^r^ (pET29a). Express *A. brasilense* G8AG60 (transcription termination factor Rho) Rho | This work |
| pFG29-GlnE | Km^r^ (pET29a). Express *A. brasilense* A7Y9V0 (Bifunctional glutamine synthetase adenylyltransferase/adenylyl-removing enzyme) ATase | This work |
| pFG29-AF97 | Km^r^ (pET29a). Express *A. brasilense* G8AF97 (Coenzyme A biosynthesis bifunctional protein) CoaBC | This work |
| pFG29-AWC0 | Km^r^ (pET29a). Express *A. brasilense* G8AWC0 (Putative signal transduction histidine kinase) | This work |
| pFG29-AKW1 | Km^r^ (pET29a). Express *A. brasilense* G8AF97 (putative hydrolase of the metallo-beta-lactamase) Ribonuclease J | This work |
| pFG29-SpeE | Km^r^ (pET29a). Express *A. brasilense* G8AGC5 (Polyamine aminopropyltransferase) SpeE | This work |
| pFG29-3o | Km^r^ (pET29a). Express *A. brasilense* G8ASZ7 (3-oxoacyl-Acyl carrier protei) reductase) | This work |
| pFG29-Dered | Km^r^ (pET29a). Express *A. brasilense* G8AWA1 (Short-chain dehydrogenase/reductase) | This work |
| pLHpet29abPTSI | Km^r^ (pET29a). Express *A. brasilense* G8AZB2 (Phosphoenolpyruvate-protein phosphotransferase) EI | This work |
| pLHpet29abRpoZ | Km^r^ (pET29a). Express *A. brasilense* G8APW6 (Uncharacterized protein DNA-directed RNA polymerase subunit omega) RpoZ) | This work |

**REFERENCES**

1. Pedrosa FO, Yates MG. 1984. Regulation of nitrogen fixation (nif) genes of Azospirillum brasilense by nifA and ntr (gln) type gene products. FEMS Microbiol Lett 23:95–101.

2. De Zamaroczy M. 1998. Structural homologues P(II) and P(z) of Azospirillum brasilense provide intracellular signalling for selective regulation of various nitrogen-dependent functions. Mol Microbiol.

3. Rocco CJ, Dennison KL, Klenchin VA, Rayment I, Escalante-Semerena JC. 2008. Construction and use of new cloning vectors for the rapid isolation of recombinant proteins from Escherichia coli. Plasmid.

4. Araujo MS, Baura VA, Souza EM, Benelli EM, Rigo LU, Steffens MBR, Pedrosa FO, Chubatsu LS. 2004. In vitro uridylylation of the Azospirillum brasilense N-signal transducing GlnZ protein. Protein Expr Purif.

5. Huergo LF, Filipaki A, Chubatsu LS, Yates MG, Steffens MB, Pedrosa FO, Souza EM. 2005. Effect of the over-expression of PII and PZ proteins on the nitrogenase activity of Azospirillum brasilense 253:47–54.

6. Huergo LF, Araújo GA, Santos AS, Gerhardt ECM, Pedrosa FO, Souza EM, Forchhammer K. 2020. The NADP-dependent malic enzyme MaeB is a central metabolic hub controlled by the acetyl-CoA to CoASH ratio. BBA - Proteins Proteomics 140462.

7. Santos ARS, Gerhardt ECM, Moure VR, Pedrosa FO, Souza EM, Diamanti R, Högbom M, Huergo LF. 2018. Kinetics and structural features of dimeric glutamine-dependent bacterial NAD synthetases suggest evolutionary adaptation to available metabolites. J Biol Chem 293.
